# Design, Synthesis and Biological Activity Evaluation of S-Substituted 1*H*-5-Mercapto-1,2,4-Triazole Derivatives as Antiproliferative Agents in Colorectal Cancer

**DOI:** 10.3389/fchem.2018.00373

**Published:** 2018-08-23

**Authors:** Marius Mioc, Sorin Avram, Vasile Bercean, Ludovic Kurunczi, Roxana M. Ghiulai, Camelia Oprean, Dorina E. Coricovac, Cristina Dehelean, Alexandra Mioc, Mihaela Balan-Porcarasu, Calin Tatu, Codruta Soica

**Affiliations:** ^1^Faculty of Pharmacy, ‘Victor Babes’ University of Medicine and Pharmacy, Timisoara, Romania; ^2^Department of Computational Chemistry, Institute of Chemistry Timisoara of the Romanian Academy, Timisoara, Romania; ^3^S.C. SINOFIN S.R.L., Timisoara, Romania; ^4^“Pius Brinzeu” Timisoara County Emergency Clinical Hospital, Oncogen Institute, Timisoara, Romania; ^5^Institute of Macromolecular Chemistry ‘Petru Poni ’, Iasi, Romania

**Keywords:** 1, 2, 4-triazole, colon cancer, antiproliferative, cell cycle, docking

## Abstract

Colon cancer is a widespread pathology with complex biochemical etiology based on a significant number of intracellular signaling pathways that play important roles in carcinogenesis, tumor proliferation and metastasis. These pathways function due to the action of key enzymes that can be used as targets for new anticancer drug development. Herein we report the synthesis and biological antiproliferative evaluation of a series of novel S-substituted 1*H*-3-R-5-mercapto-1,2,4-triazoles, on a colorectal cancer cell line, HT-29. Synthesized compounds were designed by docking based virtual screening (DBVS) of a previous constructed compound library against protein targets, known for their important role in colorectal cancer signaling: MEK1, ERK2, PDK1, VEGFR2. Among all synthesized structures, TZ55.7, which was retained as a possible PDK1 (phospholipid-dependent kinase 1) inhibitor, exhibited the most significant cytotoxic activity against HT-29 tumor cell line. The same compound alongside other two, TZ53.7 and TZ3a.7, led to a significant cell cycle arrest in both sub G0/G1 and G0/G1 phase. This study provides future perspectives for the development of new agents containing the 1,2,4-mercapto triazole scaffold with antiproliferative activities in colorectal cancer.

## Introduction

**Figure 1:**
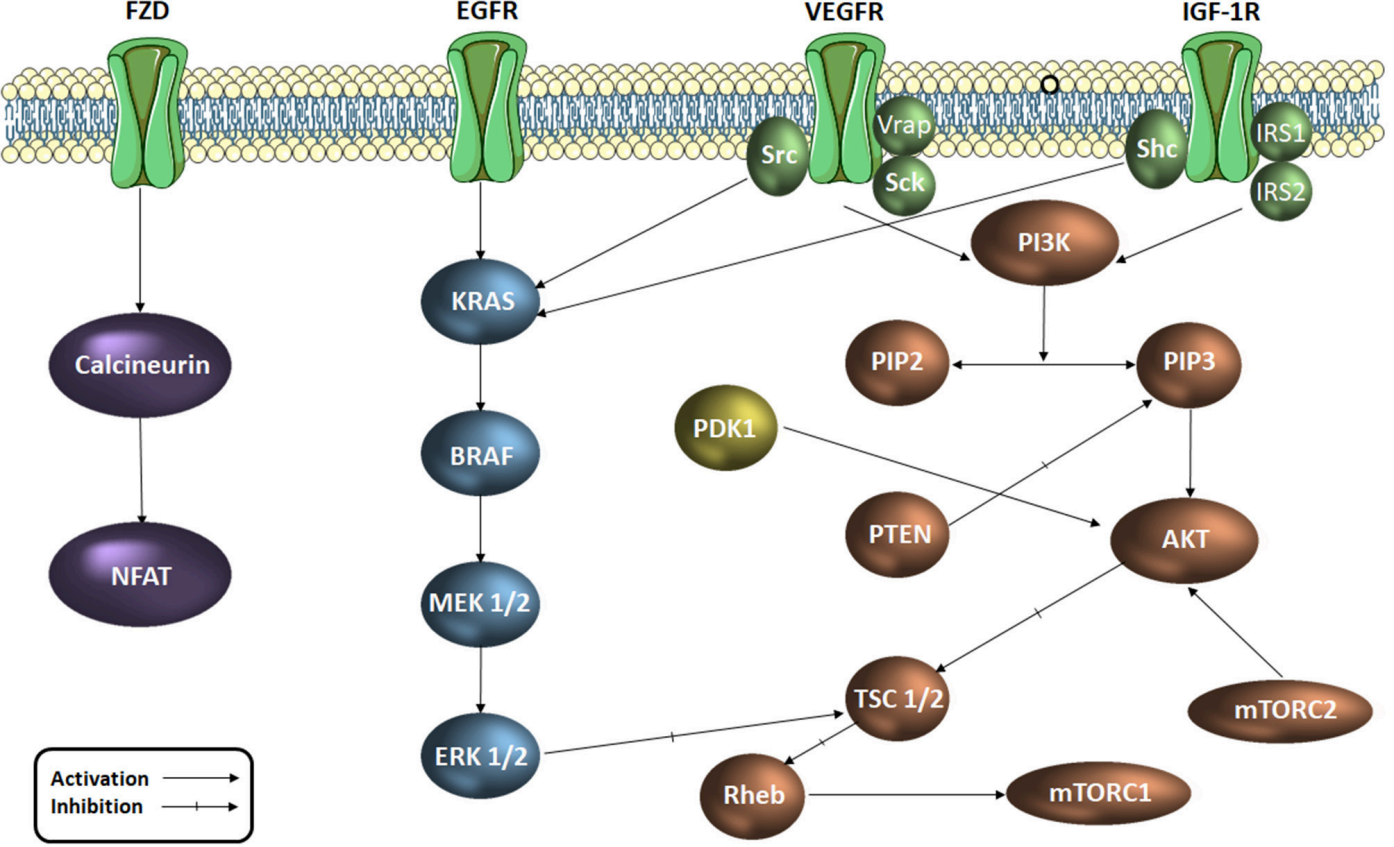
Signaling pathways in colorectal cancer (simplified scheme). AKT, protein kinase B; RAF, serin/treonin-protein kinase proto-oncogenă; RAS, retrovirus-associated DNA sequences; EGFR, epidermal growth factor receptor; ERK, extracellular regulated kinase; FZD, frizzled receptor; IGF-1R, insulin growth factor receptor 1; IRS, insulin receptor substrate; MAPK, mitogen activated protein kinase Mtorc, mammalian target of rapamycin complex; NFAT, factorul nuclear de activare a celulelor T; PDK1, phospholipid-dependent kinase 1; PI3K, phosphatidylinositol 3-kinaze; PIP2, fosfatidil-inozitol-difosfat; PIP3, fosfatidil-inozitol-trifosfat; PTEN, Phosphatase and tensin homolog; Rheb, Ras homolog enriched in brain; TSC, tuberous sclerosis protein; Shc, SHC-transforming proteins, VEGFR, vascular endothelial growth factor receptor; Vrap, VEGF Receptor-Associated Protein.

Colorectal cancer was reported as the third most common diagnosed malignancy worldwide and occupies the fourth place in the hierarchy of cancer-related deaths (Arnold et al., 2017). A significant number of signaling pathways play an important role in cell proliferation, cell cycle regulation and apoptosis at tumor level. Signaling pathways such as EGF (epidermal growth factor), VEGF (vascular endothelial growth factor), PI3K/AKT/mTOR (phosphatidylinositol 3-kinase/protein kinase B/mammalian target of rapamycin) and MAPK (mitogen activated protein kinases) support the transmission of intracellular signals which regulate and maintain cellular functions such as angiogenesis, migration, differentiation and cell proliferation (Cavasotto et al., 2006; Chen et al., 2006; Han et al., 2014). These signaling pathways (Figure 1) function through a series of enzymes and receptors that ensure signal transmission and the occurrence of the final biological outcome (Vignot et al., 2005; Angiolini et al., 2010; Han et al., 2014; Robarge et al., 2014). Active key proteins in the above mentioned pathways, with an important role in transmitting these intracellular signals, such as EGFR1 (epidermal growth factor receptor 1), VEGFR2 (endothelial vascular endothelial growth factor receptor 2), PI3Kα, AKT, MEK1 (MAP kinase/ERK kinase 1), ERK2 (extracellular regulated signaling kinase 2) and PDK1 (phospholipid-dependent kinase 1) are currently extensively studied as targets for novel drug discovery in the treatment of various types of cancer. The important part these intermediaries play within the carcinogenesis process justifies their selection as targets for the development of new anticancer agents.

According to the American Cancer Society, current targeted therapy in the treatment of colorectal cancer is quite limited to the following anticancer agents (American Cancer Society, 2017): VEGF inhibitors –bevacizumab, ramucirumab, Ziv-aflibercept; EGFR inhibitors –cetuximab, panitumumab; Kinase inhibitors: regorafenib.

The main drawback in the development of targeted therapy is that when a single signaling pathway is inhibited, cancer survival may still be ensured through the consecutive activation of alternative signaling pathways (Temraz et al., 2015).

Since its inception in the 80's, computer-aided drug design has become an important tool in discovering new molecules with particular pharmacological effects (Osakwe and Rizvi, 2016). The method uses the information provided by the tridimensional structure of a protein in order to develop new active drugs and represents a successful strategy involved in academic research and pharmaceutical industry around the world.

In the last decades 1,2,4-triazole derivatives have emerged as therapeutic drugs due to their various biological activities, including antiinflammatory and antiproliferative (Tahlan et al., 2017). The 1,2,4-triazole ring provides satisfactory water solubility, flexibility and the potential to interact with certain enzymes involved in cancer development through its ion chelating properties and the ability to form hydrogen bonds (HBs) with receptors due to the -NH- group (Aliabadi et al., 2016). Furthermore, the presence of a triazole moiety induces higher liver microsomal stability and anticancer efficacy (Kommagalla et al., 2014).

Several compounds bearing the 1,2,4-triazole scaffold were the subject of molecular docking revealing a strong interaction with the ATP-active site of tubulin through the formation of two HBs with Thr179 and Ser178 fragments (El-Sherief et al., 2018). Their antiproliferative activity was tested against four cancer cell lines including HT-29 (colon cancer) revealing IC_50_ values in the micromolar range (El-Sherief et al., 2018). Another series of 1,2,4-triazole derivatives bearing the dimethylaminoethyl group was synthesized by Qin et al. in 2014 and tested as antiproliferative agents against five cancer cell lines including the HT-29 (human colorectal cancer); *in vitro* tests revealed that all compounds exerted a strong yet selective cytotoxic activity against the colon cancer cell line acting as potential Raf kinase inhibitors (Qin et al., 2014). N-(5-benzylthio)-4H-1,2,4-triazol-3-yl)-4-fluorobenzamide derivatives were synthesized by Aliabadi et al. in 2016 by means of bioisosteric replacement; all tested compounds proved most active against the HT-29 colon cancer cell line, two molecules exhibiting even higher antiproliferative activities compared to imatinib, the reference anticancer drug (Aliabadi et al., 2016).

**Figure 2:**
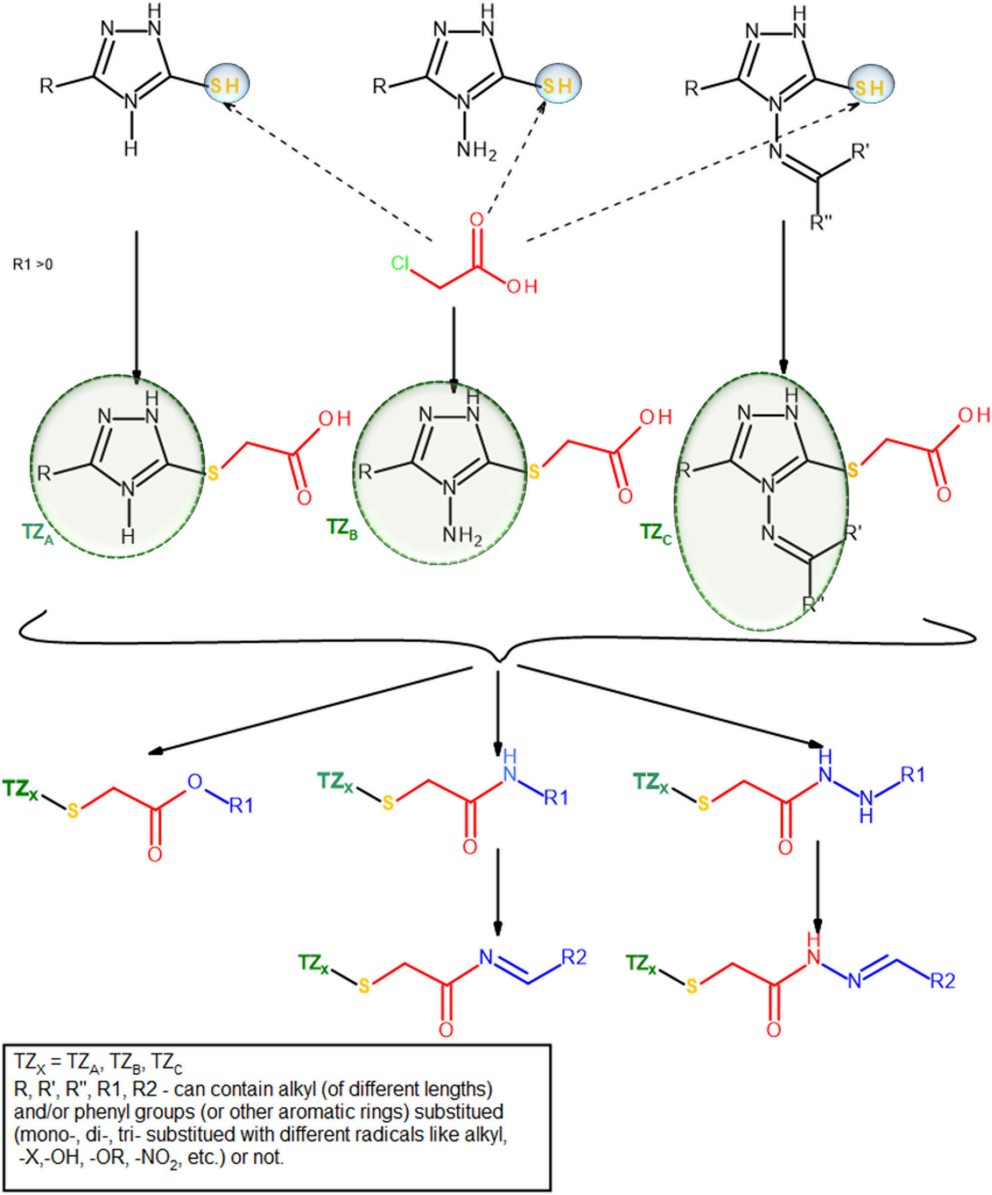
Compound library construction scheme.

The group of Bercean et al. synthesized a large body of 5-mercapto-1,2,4 triazoles, some of which being previously reported in the literature (Bercean et al., 2003, 2010; Lascu et al., 2010; Ledeti et al., 2010). Unpublished data regarding the *in vitro* anticancer activity of some of these compounds showed low to moderate effects when tested on certain types of cancer cell lines. Other studies showed that S-alkylated mercapto-1,2,4-triazoles, bearing large substituents which contain HB donor/acceptor atoms, such as nitrogen (alkyl-amino or N-substituted alkyl-pyperazinyl), exhibited significant anticancer activity on the tested cancer cell lines, including colon cancer (Murty et al., 2012; Philip et al., 2014). Consequently, we built a small molecule library, previously reported in another study (Mioc et al., 2017c) using the available 5-mercapto-1,2,4-triazole as scaffold, by attaching various radicals containing nitrogen and oxygen atoms (Figure 2), for the purpose of docking based virtual screening (DBVS) and subsequent synthesis of hit molecules with potential anticancer activity.

In the current study we report the synthesis and antiproliferative activity evaluation of a series of novel S-substituted 1*H*-3-R-5-mercapto-1,2,4-triazoles selected trough DBVS of a previously described compound library.

## Materials and methods

Ethanol, *N,N*-dimethylformamide, pyridine, ethyl chloroacetate, chloroacetamide were purchased from Sigma Aldrich, (St. Louis, MO, USA) and used without further purification. N-benzylideneamino-2-chloro-acetamide, was synthesized according to literature data and used without further purification. Melting points were carried out on a Böetius PHMK (Veb Analytik Dresden) apparatus; thin-layer chromatography (TLC) was employed on silica gel-coated plates 60 F254 Merck using various ratios of hexane: ethyl acetate, as eluents. IR spectra were recorded on KBr pellets on a Jasco FT/IR-410 spectrophotometer. NMR spectra were recorded on a Bruker Avance DRX 400 spectrometer using DMSO-d6 as solvent (room temperature). Chemical shifts are reported as δ values (ppm) and were referenced to the solvent's residual peak (2.51 ppm for ^1^H and 39.47 ppm for ^13^C). Mass spectra analysis was conducted on a Agilent6120 Quadrupole LC/MS system (Santa Clara, CA USA) equipped with a UV detector, ESI ionization source and a Zorbax Rapid Resolution SB-C18 **(**1.8 μm; 50 × 2.1 mm) column. LC/MS spectra of all samples were carried out using methanolic solutions of the compounds tested. All samples were analyzed using LC/MS grade methanol (Merck, Darmstadt, Germany) as isocratic mobile phase, at a flow rate of 0.4 mL/min, temperature of 25°C and λ = 250 nm. Mass spectra were recorded in the positive ion mode using optimized ESI parameters: nitrogen nebulizer pressure, 35psi, nitrogen drying gas temperature, 250°C, flow rate at 12 L/min and capillary voltage set at 3,000 V, in the Scan mode. Elemental analysis was done using a Vario EL Elementar Analysensysteme GmbH.

### Molecular docking

For the purpose of the current study, a created compound library containing 5-mercapto-1,2,4-triazole derivatives, described in a previous work (Mioc et al., 2017b,c), was virtually screened against protein targets, known for their important role in colorectal cancer signaling: MEK1, ERK2, PDK1, VEGFR2.

For molecular docking, the HYBRID software, version 3.0.1 (OpenEye Scientific Software, Inc.) was used (McGann, 2012). The protein selection/preparation and docking method employed was also described in a previous work (Mioc et al., 2017b,c).

The 3D structures, corresponding to the selected protein targets (Table 1), were obtained from the RCSB PDB (Research Collaborative for Structural Bioinformatics Protein Data Bank) and processed with the OEDocking MAKE_RECEPTOR software, version 3.0.1 (OpenEye Scientific Software, Inc.) (Hawkins et al., 2010). Protein structures for each target, were selected following three requirements: (1) protein structures containing a co-crystalized ligand (requirement necessary for the docking software); (2) non-mutant variations of protein structures; (3) protein structures with the Cruickshank DPI (diffraction precision index) <0.5 (Gurusaran et al., 2014). The preparation of protein structures, using the MAKE_RECEPTOR software, was a multi-step process in which, in the first phase, from the uploaded protein-ligand complex file, the protein backbone and the co-crystallized ligand were defined as such and water molecules were removed. Next, an adjustable grid box (later used as the docking space) was built around the co-crystallized ligand, after which a search has been performed to detect cavities from the binding site, using one of two possible program embedded algorithms (molecular or atomic). The boundaries of the above mentioned generated grid could be further adjusted to incorporate a possible adjacent discovered cavity. In the next step the program calculates a potential surface for the ligand, delimited by two contours (inner and outer), boundaries used in docking, in the similarity search phase between the co-crystallized ligand and the docked compounds. In this case the outer contour was used in molecular docking so that docked compound conformations that reached this outer line were discarded. Finally the structure was exported in a suitable file format for the docking software.

**Table 1:** PDB ID's of the selected 3D protein structures used as targets for virtual screening.

**Protein target**	**3D structure PDB ID**
MEK1	1S9J, 3DY7, 3E8N, 3EQB, 3EQC, 3EQG, 3EQF, 3EQH, 3MB1, 3ORN, 3OS3, 3PP1, 3SLS, 3V01, 3V04, 3W8Q, 3WIG, 3ZLS, 3ZLW, 3ZLX, 3ZLY,3ZM4, 4AN3, 4AN9, 4ANB, 4ARK, 4U7Z, 4U80, 4U81
ERK2	2OJG, 2Y9Q, 3SAO, 3I5Z, 3W55, 4FV0, 4FV1, 4FV2, 4FV3, 4FV5, 4FV7, 4FV8, 4FV9, 4FUX, 4FUY, 4FMQ, 4G6N, 4NOS, 4QTA, 4QTE, 4O6E, 4G6O, 4H3Q
PDK1	1OKY, 1UVR, 1OKZ, 1UU3, 1UU7, 1UU8, 1UU9, 1Z5M, 2PE0, 2PE1, 2PE2, 2R7B, 2XCH, 2XCK, 3H9O, 3ION, 3IOP, 3NAX, 3NUN, 3NUU, 3NUY, 3PWY, 3QCQ, 3QCS, 3QCX, 3QCY, 3QD0, 3QD3, 3QD4, 3RCJ, 3RWP, 3RWQ, 4A06, 4A07
VEGFR2	1Y6A, 1Y6B, 1YWN, 2OH4, 2P2H, 2QU5, 2RL5, 2XIR, 3C7Q, 3CJG, 3CJF, 3EWH, 3U6J, 3VHE, 3VHK, 3VID, 3VNT, 3VO3, 4AG8, 4AGC, 4AGD, 4ASD, 4ASE

The compound library was screened against each set of 3D protein structures corresponding to a certain protein target (**Table 4**). All molecules were docked in the ATP-binding site of each target, except MEK1 for which the adjacent binding pocket, next to the ATP binding site was used. Docked molecules were ranked according to the docking scores (Chemgauss 4) attributed by the software's scoring function (McGann, 2012). Docking results interpretation was carried out using Discovery Studio 4.1 (Dassault Systemes, BIOVIA Corp., San Diego, CA, USA).

After molecular docking, since the docking program has a ligand-based scoring function, from all 3D protein structure sets, corresponding to a specific target, the protein structure with the highest docked molecule number was retained for further analysis, meaning that the respective protein binding site (or binding site conformation) is the most adequate for later receptor-target binding analysis. As such the following protein structures were selected for further analysis: MEK1- PDB ID: 3PP1; ERK2- PDB ID: 4G6N; PDK1- PDB ID: 2PE1, VEGFR2 – PDB ID: 3VHK. From each docked molecules score list, the first 50 molecules, ranked by the docking score, were analyzed, taking into account their interactions with the binding site residues and the topological similarity with each co-crystalized ligand of the protein.

The compound selection workflow was described in a previous study (Mioc et al., 2017b). Briefly, a similarity analysis between the binding pattern of the first 50 ranked docked compounds and the co-crystalized ligand for each target protein, was carried out, in terms of the number and type of interactions formed as well as the aminoacid residues involved. Next a preliminary selection was made for each target, after which the selected set was once more visually analyzed in terms of the overall spatial orientation similarity with the crystallized ligand and the key interactions formed within the binding site. After this last step, the final compound selection proposed for chemical synthesis was obtained.

### Chemistry

**Figure 3:**
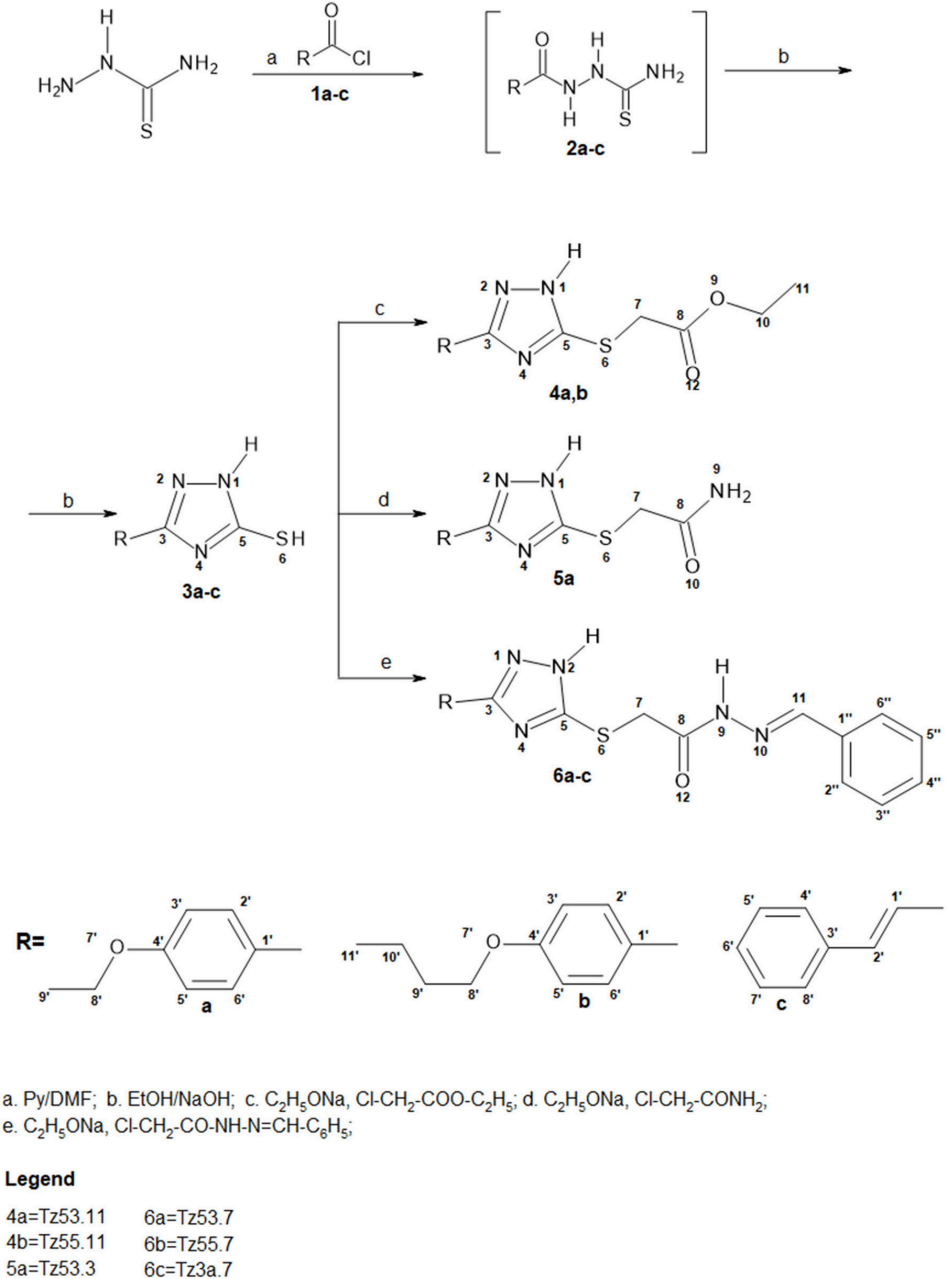
Synthesis pathway of S-functionalized 1H-3-R-5-mercapto-1,2,4-triazoles.

Synthesis pathway of S-functionalized 1*H*-3-R-5-mercapto-1,2,4-triazoles is depicted in Figure 3. The procedure used 1*H*-3-R-5-mercapto-1,2,4-triazoles (3a-c) as starting materials, which were synthesized using a previously described method (Bercean et al., 2003) and were previously reported in another study (Mioc et al., 2017c). Briefly, the synthesis of compounds 3a-c was accomplished by thiosemicarbazide acylation with the corresponding acyl chlorides (1a-c) in N,N-Dimethylformamide (DMF), in the presence of pyridine, followed by cyclization of the unpurified 1-acyl-thiosemicarbazides (2a-c) in ethanolic NaOH, at reflux.

#### General synthesis procedure for all compounds

Starting compound materials (1a-c) (1 mmol) were dissolved, under stirring, in a 10 ml ethanolic solution of Na (230 mg, 1mmol). A colorless solution was obtained. After 1 min, 1 mmol (1 equiv) of alkylating agent was added, after which the immediate formation of a white precipitate was observed. The solution was maintained under stirring for about 3h at room temperature. Formation of the compounds was verified by TLC, using n-hexane:ethyl acetate (He:EA), 1:1 as eluent, until consumption of the reactants was observed. The reaction products were purified by water precipitation and repeated extractions with ethyl acetate (EA) until a single TLC spot appeared, after which the extracted fractions were dehydrated with anhydrous Na_2_SO_4_ and the solvent was evaporated to obtain the pure product. In the case of compounds TZ53.7 and TZ3a.7, the reactants were not fully consumed as revealed by TLC, and thus couldn't be purified by the above mentioned method since the intermediates used as starting material were also soluble in EA. Consequently they were purified by flash column chromatography using a He:EA mixture of 1:1 (v/v) as eluent. Fractions containing only the reaction product were evaporated to obtain the pure final product. The compounds were characterized by elemental analysis, IR spectroscopy, NMR and mass spectrometry coupled with liquid chromatography (LC-MS). Compound characterization values are listed below. The original spectra of all synthesized compounds are available in the Supplementary material published with the present paper. Characterization and synthesis of compound TZ3a.7 was reported in a previous work (Mioc et al., 2017a).

### 1H-3-(4-ethoxiphenyl)-5-aminocarbonyl-metylsulfanyl-1,2,4-triazole (5a) (tz53.3)

White cristalyne powder; Yield: 68%; m.p. = 195–196°C (EtOH); TLC: (Tol:MeOH = 2:1) *R*_*f*_ = 0.69; ^1^H-RMN, 400.13 MHz, δ (ppm), DMSO-d_6_: 14.26 (s, br, H1), 7.88 (d, *J* = 8.6 Hz, 2H, H2′,6′), 7.63, 7.21 (2H, H9), 7.05 (d, *J* = 6.7 Hz, 2H, H3′,5′), 4.08 (q, *J* = 6.9 Hz, 2H, H8′), 3.86 (s, 2H, H7), 1.34 (t, *J* = 6.9 Hz, 3H, H9′); ^13^C-RMN, 100.6 MHz, δ (ppm), DMSO-d_6_: 169.5 (C8), 160.1 (C4′), 159.2 (C5), 155.2 (C3), 127.7 (C2′, C6′), 119.1 (C1′), 114.9 (C3′, C5′), 63.3 (C8′), 33.2 (C7), 14.6 (C9′); IR [KBr] (cm^−1^): 3,392, 3,323, 3,186, 3,060, 2,979, 2,968, 2,920, 2,896, 1,656, 1,615, 1,502, 1,474, 1,447, 1,408, 1,392, 1,323, 1,290, 1,257, 1,232, 1,182, 1,147, 1,119, 1,042, 979, 920, 846, 823, 746, 661, 641, 541; Elemental analysis: calculated values for C12H14N4O2S: C, 51.78; H, 5.07; N, 20.13; experimental values: C, 51.39; H, 5.21; N, 21.62; LC-MS (ESI): Rt = 0.590 min; *m/z* = 279.0 [M+ H^+^]^+^.

### 1H-3-(4-ethoxifenyl)-5-benzilidenehydrazino-carbonyl-metilsulfanyl-1,2,4-triazole (6a) (tz53.7)

White crystalyne powder; Yield: 60%; m.p. = 82–90°C; TLC: (Hexan:EA = 1:1) *R*_*f*_ = 0.18; ^1^H-RMN, 400.13 MHz, δ (ppm), DMSO-d_6_:, 14.3, 11.6 (NH), 8.23, 8.03 (H11), 7.90-7.86 (H2′, H6′), 7.69 (H2′′, H6′′), 7.45-7.41 (H3′′, H4′′, H5′′), 7.04 (H3′, H5′), 4.43, 4.00 (H7), 4.01 (H8′), 1.34 (H9′); ^13^C-RMN, 100.6 MHz, δ (ppm), DMSO-d_6_: 169.5 (C8), 160.1 (C4′), 159.1 (C5), 151.2 (C3), 146.9, 143.4 (C11), 134.1 (C1′′), 130.2, 129.9 (C4′′), 128.8 (C3′′, C5′′), 127.7 (C2′, C6′), 127.1, 126.9 (C2′′, C6′′), 119.1 (C1′), 114.9 (C3′, C5′), 63.3 (C8′), 34.2, 33.2 (C7), 14.6 (C9′); IR [KBr] (cm^−1^): 3,464, 3,187, 3,098, 3,064, 3,031, 2,980, 2,934, 2,882, 1,670, 1,614, 1,580, 1,502, 1,450, 1,394, 1,330, 1,292, 1,254, 1,179, 1,138, 1,044, 983, 922, 841, 756, 692, 660, 510; Elemental analysis: calculated values for C19H19N5O2S: C, 59.83; H, 5.02; N, 18.36; experimental values: C, 59.54; H, 5.17; N, 18.18; LC-MS (ESI): Rt = 0.623 min; *m/z* = 382.0 [M+ H^+^]^+^.

### 1H-3-(4-n-butoxifenyl)-5-benzilidenehydrazino-carbonyl-metilsulfanyl-1,2,4-triazole (6b) (tz55.7)

White crystalyne powder; Yield: 60%; m.p. = 83–90°C; TLC: (Hexan:EA = 1:1) *R*_*f*_ = 0.14; ^1^H-RMN, 400.13 MHz, δ (ppm), DMSO-d_6_: 11.6 (NH), 8.23, 8.03 (H11), 7.90–7.86 (H2′, H6′), 7.68–7.67 (H2′′, H6′′), 7.44-7.41 (H3′′, H4′′, H5′′), 7.02 (H3′, H5′), 4.44, 3.99 (H7), 4.01 (H8′), 1.70 (H9′), 1.44 (H10′), 0.93 (H11′); ^13^C-RMN, 100.6 MHz, δ (ppm), DMSO-d_6_: 169.5, 164.4 (C8), 160.0, 159.9 (C4′), 157.1, 157.0, 156.8, 156.7 (C3, C5), 146.9, 143.4 (C11), 134.1, 134.0 (C1′′), 130.1, 129.9 (C4′′), 128.8 (C3′′, C5′′), 127.5 (C2′, C6′), 127.1, 126.8 (C2′′, C6′′), 120.6, 120.3 (C1′), 114.8, 114.7 (C3′, C5′), 67.3 (C8′), 34.5, 33.4 (C7), 30.7 (C9′), 18.7 (C10′), 13.7 (C11′); IR [KBr] (cm^−1^):3,445, 3,200, 3,105, 3,067, 2,958, 2,933, 2,872, 1,670, 1,614, 1,578, 1,501, 1,450, 1,395, 1,291, 1,254, 1,178, 1,138, 1,066, 983, 838, 756, 692, 661, 510; Elemental analysis: calculated values for C_21_H_23_N_5_O_2_S: C, 61.59; H, 5.66; N, 17.10; experimental values: C, 61.64; H, 5.62; N, 16.98; LC-MS (ESI): Rt = 0.659 min; *m/z* = 410.0 [M+ H^+^]^+^.

### 1H-3-(4-ethoxifenyl)-5-ethoxicarbonyl-metilsulfanyl-1,2,4-triazole(4a) (tz 53.11)

White powder; Yield: 72%; m.p. = 120–121°C, TLC: (He:EA = 1:1) *R*_*f*_ = 0.51; ^1^H-RMN, 400.13 MHz, δ (ppm), DMSO-d_6_: 7.86 (d, *J* = 8.4 Hz, 2H, H2′,6′), 7.05 (d, *J* = 8.4 Hz, 2H, H3′,5′), 4.15-4.06 (4H, H8′, H10), 4.04 (s, 2H, H7), 1.34 (t, *J* = 6.8 Hz, 3H, H9′), 1.18 (t, *J* = 7.0 Hz, 3H, H11); ^13^C-RMN, 100.6 MHz, δ (ppm), DMSO-d_6_: 168.8 (C8), 160.0 (C4′), 156.3 (C3, C5), 127.6 (C2′, C6′), 119.8 (C1′), 114.8 (C3′, C5′), 63.3 (C8′), 61.0 (C10), 33.5 (C7), 14.6 (C9′), 14.0 (C11); IR [KBr] (cm^−1^):3,160, 3,109, 3,075, 3,034, 2,977, 2,933, 2,885, 1,737, 1,715, 1,615, 1,585, 1,502, 1,474, 1,393, 1,332, 1,307, 1,291, 1,268, 1,194, 1,181, 1,168, 1,130, 1,045, 1,001, 926, 840, 743, 715, 660, 520; Elemental analysis: calculated values for C_14_H_17_N_3_O_3_S: C, 54.71; H, 5.58; N, 13.67; experimental values: C, 54.12; H, 5.66; N, 13.58; LC-MS (ESI): Rt = 0.660 min; *m/z* = 308.0 [M+ H^+^]^+^.

### 1H-3-(4-n-butoxiphenyl)-5-ethoxicarbonyl-metilsulfanyl-1,2,4-triazole (4b) (TZ 55.11)

White crystalyne powder; Yield: 78%; m.p. = 120–121°C (EtOH); TLC: (He:EA = 1:1) *R*_*f*_ = 0.49; ^1^H-RMN, 400.13 MHz, δ (ppm), DMSO-d_6_: 14.25 (s, br, H1), 7.86 (d, *J* = 8.6 Hz, 2H, H2′,6′), 7.06 (d, *J* = 8.5 Hz, 2H, H3′,5′), 4.12 (q, *J* = 7.1 Hz, 2H, H10), 4.04–4.00 (4H, H8′, H7), 1.71 (m, *J* = 6.9 Hz, 2H, H9′), 1.44 (m, *J* = 7.4 Hz, 2H, H10′), 1.18 (t, *J* = 7.1 Hz, 3H, H11), 0.93 (t, *J* = 7.4 Hz, 3H, H11′); ^13^C-RMN, 100.6 MHz, δ (ppm), DMSO-d_6_: 168.8 (C8), 160.2 (C4′), 157.2 (C3, C5), 127.6 (C2′, C6′), 119.4 (C1′), 114.9 (C3′, C5′), 67.4 (C8′), 61.0 (C10), 33.5 (C7), 30.1 (C9′), 18.7 (C10′), 14.0 (C11), 13.7 (C11′); IR [KBr] (cm^−1^): 3,163, 3,101, 3,065, 3,027, 3,006, 2,960, 2,936, 2,909, 2,871, 1,739, 1,717, 1,616, 1,587, 1,504, 1,471, 1,390, 1,332, 1,303, 1,289, 1,265, 1,190, 1,179, 1,167, 1,135, 1,047, 1,004, 925, 842, 745, 715, 666, 519; Elemental analysis: calculated values for C_16_H_21_N_3_O_3_S: C, 57.29; H, 6.31; N, 12.53; experimental values: C, 57.16; H, 6.86; N, 12.44; LC-MS (ESI): Rt = 0.659; *m/z* = 336.0 [M+ H^+^]^+^.

### Biological evaluation

#### Cell culture

HT-29 colorectal adenocarcinoma cells were grown according to the protocol as follows: after rapid thawing at 37°C, the cells were centrifuged at 1500 RPM, 25°C for 7 min in the culture medium, preheated at 37°C. The cell pellet thus obtained was resuspended in the medium cultured in 75 cm^2^ culture plates in a humidified thermostated incubator with 5% CO_2_ at 37°C. The culture medium specific for the HT-29 line is McCoy's 5A Medium (ATCC), 1.5 mM L-glutamine and 2,200 mg/L sodium bicarbonate supplemented with 10% fetal calf serum (FCS; PromoCell) and 1% Penicillin/Streptomycin (Pen/Strep, 10,000 IU/ml; PromoCell) mixture. The cells were grown, and the culture medium was renewed for 3–4 days to a confluency of 80–90% when the passage was made. Human keratinocytes (HaCaT) were a gift from the University of Debrecen, Debrecen, Hungary. The cells were cultured in Dulbecco's modified Eagle's Medium (DMEM) containing 4.5 g/l glucose, L-glutamine and NaHCO3, and supplemented with 100 U/ml penicillin, 100 μg/ml streptomycin and 10% fetal bovine serum (FBS). Cells were kept under standard conditions (humidified atmosphere, 5% CO_2_, 37°C) and passaged every second day.

#### *In vitro* cell viability evaluation—Alamar Blue assay

The HT-29 cell line was cultured in the specific culture medium according to the above mentioned protocol. The cells were seeded in a 96-well plate (10,000 cells / well) and allowed to adhere for 24 h. The next day, the culture medium was replaced with a volume of 100 μL of fresh medium containing the test substances, and the cells were incubated for 24 h. The concentrations tested were 50, 150, 200, 250, and 350 μM. After 24 h of exposure to the test compounds, 10 μL of Alamar Blue reagent (10% of the initial volume) was added to each well and the plates were incubated at 37°C for 6 h when the color reaction occurred; the absorbance was read at 570 and 600 nm using a Tecan Infinite 200 Pro reader microplate reader. All samples were executed in quadruplicate. The untreated cells, introduced into the experiment with normal proliferation medium only, were used as controls. Since stock solutions were obtained by dissolving the test substances in DMSO, the effect of the solvent on the cells was also measured at a concentration of 0.35% (maximum concentration of DMSO in the samples).

#### Determination of VEGFR2 secreted by HT-29 cells into culture media using enzyme-linked immunosorbent assay (ELISA)

HT-29 cells (1 × 10^4^ cells/well/200 μL) were seeded in a 96-well plate and stimulated for 24 h with the test compounds: TZ55.7 (50 and 100 μM), TZ53.7 (150 and 250 μM) and TZ3a.7 (150 and 250 μM) for 24h. After 24 h, the cells debris were removed by centrifugation and the cells supernatants were collected for ELISA test. The concentration of vascular endothelial growth factor (VEGFR2) in the supernatant of HT-29 cells, stimulated with test compounds (TZ55.7, TZ53.7 and TZ3a.7) was measured according to the manufacturer's protocol using Human VEGFR2/KDR Platinum ELISA commercial kit (BMS2019, eBioscience, Bender MedSystems GmbH, Austria). In brief, 50 μL of each sample supernatant were added to a 96-well microplate pre-coated with VEGFR2 antibody and incubated with anti-human VEGFR2/KDR Biotin-Conjugate for 2 h at room temperature. Then the plates were incubated with Streptavidin-HRP for 1 h at room temperature. A color reaction was obtained after adding the TMB substrate solution, reaction that was stopped after 30 min by adding the stop solution. The absorbance was measured at 450 nm. The standard curve and the quantification of the amount of VEGFR2 in the samples were obtained with GraphPad Prism 6 software. The supernatant collected from unstimulated cells was used as control. Each sample was tested in triplicate.

#### Immunofluorescence staining

To perform this test, HT-29 cells were cultured at a density of 2 × 10^6^ cells on a sterile slide in 6-wells plate and stimulated with TZ55.7 (50 and 100 μM), TZ53.7 (150 and 250 μM) and TZ3a.7 (150 and 250 μM) for 24 h. The applied method used is a slightly modified protocol, which was described in our previous studies (Gheorgheosu et al., 2013). Briefly, after the 24 h stimulation, the cells were fixed with paraformaldehyde 4% for 1h at room temperature (RT) followed by permeabilization with a 2% Triton X/PBS solution for 30 min at RT. The permeabilization was blocked by adding a 30% FBS in 0.01% Triton X for 1h and the cells were incubated with the primary antibody (1:100)—Phospho-PDK1 (Ser241) Polyclonal Antibody (Invitrogen, Germany) overnight at 4°C (dark). The secondary antibody (1:100)—AlexaFluor 488 Goat Anti-rabbit (Thermo Fisher Scientific, USA) was added the next day for 2h at RT (dark). The nucleus was also counterstained using 4′,6-diamidino-2-phenylindol (DAPI) (Sigma Aldrich, Germany) for 30 min. The slides were mounted using Fluoromount G (Biozol, Germany). Images were acquired by Olympus IX73 inverted microscope provided with DP74 camera photo, documented with the CellSens V1.15 software (Olympus, Tokyo, Japan) and were merged by ImageJ Software.

#### Cell cycle analysis

Cell cycle analysis was performed using flow cytometry. The HT-29 human colorectal carcinoma cells were seeded into 6 well plates at a density of 5 × 10^5^ cells/well. Next day, the culture medium was removed and a new medium containing the tested substances was added. Following the Alamar Blue assay and determination of IC50 values, 2 concentrations of each compound were selected in order to perform the cell cycle analysis. The selected concentrations were 50 and 100 μM for TZ55.7 (IC_50_ – 87.95 μM); 150 and 250 μM for TZ53.7 and TZ3a.7 (IC50 – 239.25 and 149.25 μM, respectively); 150 and 350 μM for the compounds with a calculated IC50 higher than the concentrations tested using Alamar Blue assay or with undetected IC50, namely TZ53.11 and TZ55.11 (IC50 – 437.84 and 374.10 μM, respectively) and TZ53.3. The final concentration of the tested compounds was obtained by successive dilutions into the culture medium starting from a stock solution of 100 mM in DMSO. Untreated cells were used as control; cells treated with DMSO were used as solvent control.

After 24 h of treatment, the cells were collected, fixed with cold 70% ethanol and stored for 30 min at 4°C. After centrifugation (1500 rpm, 5 min, 22°C) the cells were washed with cold PBS (Phosphate Buffer Saline). Subsequently, 100 μl of propidium iodide (concentration, 100 μM) (BD Pharmingen; BD Biosciences, San Diego, CA, USA) were added to the cells in order to stain the DNA and the cells were incubated for 10 min in the dark. A FACSCalibur flow cytometer (Becton-Dickinson, Franklin Lakes, NJ, USA) was used to perform the DNA content analysis. Flowing Software 2.5.1. was used to determine the percentage of cells present in the different cell cycle phases.

#### Annexin V apoptosis assay

For the flow cytometric analysis of cell apoptosis an Annexin V-FITC kit (Invitrogen, ThermoFisher, Vienna, Austria) was used. 5 × 10^5^cells/well were seeded into a 6 well plate (Greiner bio-one) and treated with the tested compounds for 48 h. Following the Alamar Blue assay and determination of IC_50_ values, 2 concentrations of each compound were selected in order to perform the Annexin V/PI analysis. The selected concentrations were 50 and 100 μM for TZ55.7 (IC50 – 87.95 μM); 150 and 250 μM for TZ53.7 and TZ3a.7 (IC_50_ – 239.25 and 149.25 μM, respectively); 150 and 350 μM for the compounds with a calculated IC_50_ higher than the concentrations tested using Alamar Blue assay or with undetected IC_50_, namely TZ53.11 and TZ55.11 (IC50 – 437.84 and 374.10 μM, respectively) and TZ53.3. The final concentration of the tested compounds was obtained by successive dilutions into the culture medium starting from a stock solution of 100 mM in DMSO. Untreated cells were used as control; cells treated with DMSO were used as solvent control. After trypsinisation, cells were collected, resuspended in 500 μL PBS (Sigma Aldrich) and centrifuged for 5 min at 1500 rpm, then the flow cytometric studies were performed following the manufacturer's protocol. Briefly, 2-5x10^5^ cells were two times washed in 1 × Annexin V Binding Buffer, centrifuged at 1500 rpm for 5 min, resuspended in the binding buffer and incubated with 5 μL of Annexin V-FITC for 15 min in the dark. After washing the cells with 200 μL specific binding buffer and centrifugation, the cell pellet was resuspended in 190 μL binding buffer, and 10 μL of PI solution was added immediately prior to analysis by flow cytometry using a FACSCalibur flow cytometer (Becton Dickinson, Franklin Lakes, NJ, USA). The results were analyzed using Flowing Software 2.5.1.

### Statistics

The results are presented as mean value ± standard deviation. One way ANOVA test was used to determine the statistical difference between various experimental groups; ^*^, ^**^, and ^***^ indicate *p* < 0.05, *p* < 0.01, and *p* < 0.001, respectively.

## Results and discussions

### Molecular docking

After carefully analyzing the accommodation of each molecule in their respective binding site, according to the final docking results, 4 molecules were retained as suitable for synthesis, namely 1*H*-3-(4-ethoxiphenyl)-5-aminocarbonyl-metylsulfanyl-1,2,4-triazole (TZ53.3), 1*H*-3-(4-ethoxifenyl)-5-benzilidenehydrazino-carbonyl-metilsulfanyl-1,2,4-triazole (TZ53.7), 1*H*-3-(4-n-butoxifenyl)-5-benzilidenehydrazino-carbonyl-metilsulfanyl-1,2,4-triazole (TZ55.7) and 1*H*-3-styryl-5-benzylidenehydrazino-carbonylmethylsulfanil-1,2,4-triazoles (TZ3a.7).

**Figure 4:**
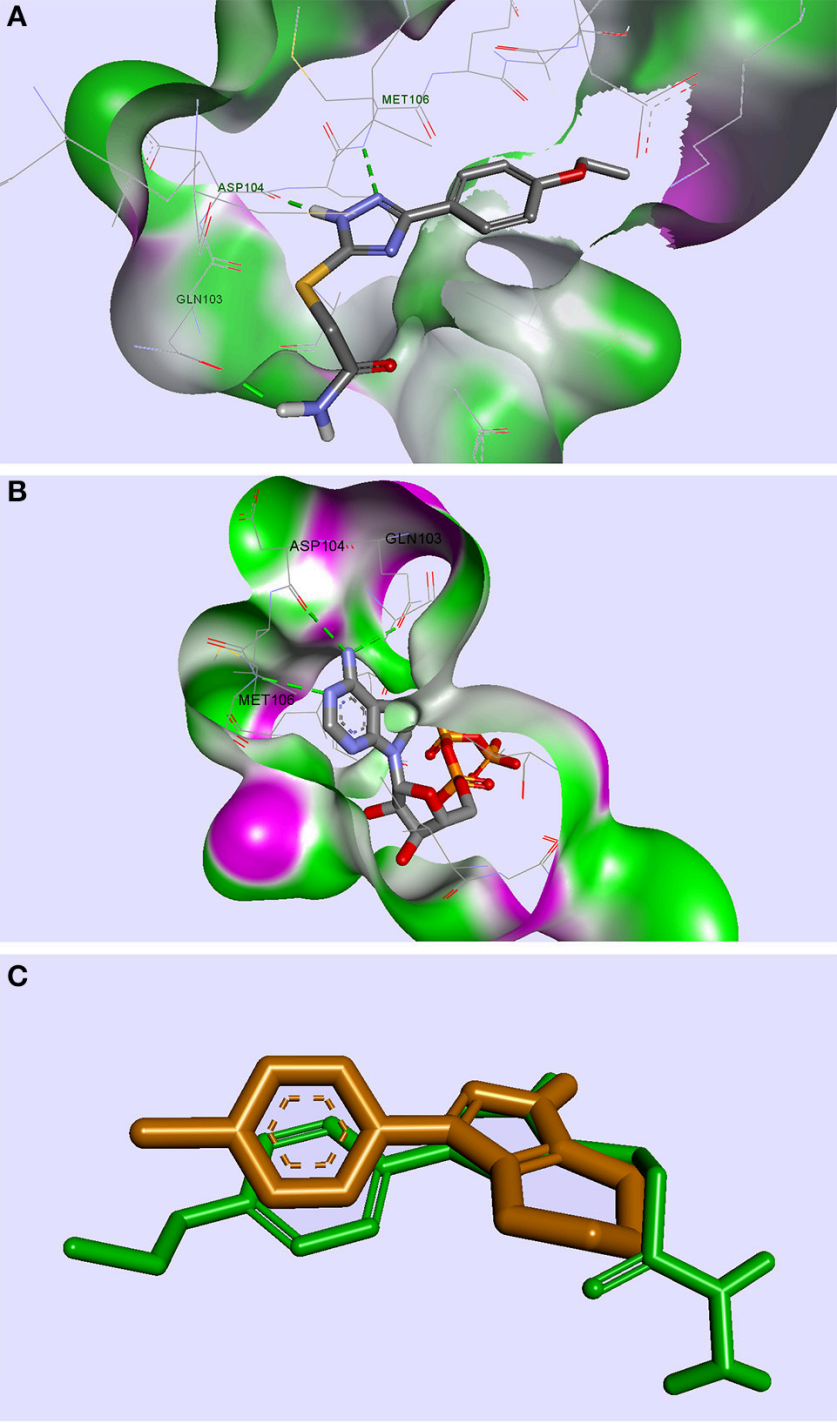
Compound TZ53.3 docked in the ATP active site of the ERK2 protein, PDB ID: 4G6N **(A)**; ERK2 protein co-crystalized with ATP molecule, PDB ID: 4GT6 **(B)**; superimposed structures of compound TZ53.3 and the co-crystalized ligand of ERK2, PDB ID: 4G6N **(C)**; HBs are depicted as green dotted lines.

From the selection corresponding to the ERK2 protein, the compound TZ53.3 proved to be well accommodated in the protein binding site. A few molecules docked in ERK2 showed the formation of 3 important HBs with the aminoacid residues Gln103, Asp104, Met106, respectively, similarly with the bound ATP molecule (Pozharski et al., 2012); however, only compound TZ53.3 exhibited a superior spatial orientation and coplanarity with the co-crystallized ligand in the active site of the ERK2 protein used (Figure 4). It appears that in this case, the observed binding pattern of each compound docked in ERK2 suggests that the triazole ring is responsible for two of the three hydrogen bridges formed (with Asp104 and Met106, respectively), while the HB with Gln103 is only present for compounds containing a HB donor atom on the S-substituted side chain.

In the case of MEK1, the compounds were docked in the binding pocket adjacent to the ATP binding site. According to Lu et al, key interactions for MEK1 non-competitive ATP inhibitors, assessed from Pfizer's PD318088 binding mode, are as follows: electrostatic interaction with Val127's carbonyl, HB with Ser212/Val211 and Lys97 (Lu et al., 2014). Following binding mode analysis none of the docked selected compounds have been shown to meet satisfactory conditions for a potential MEK1 inhibitor.

**Figure 5:**
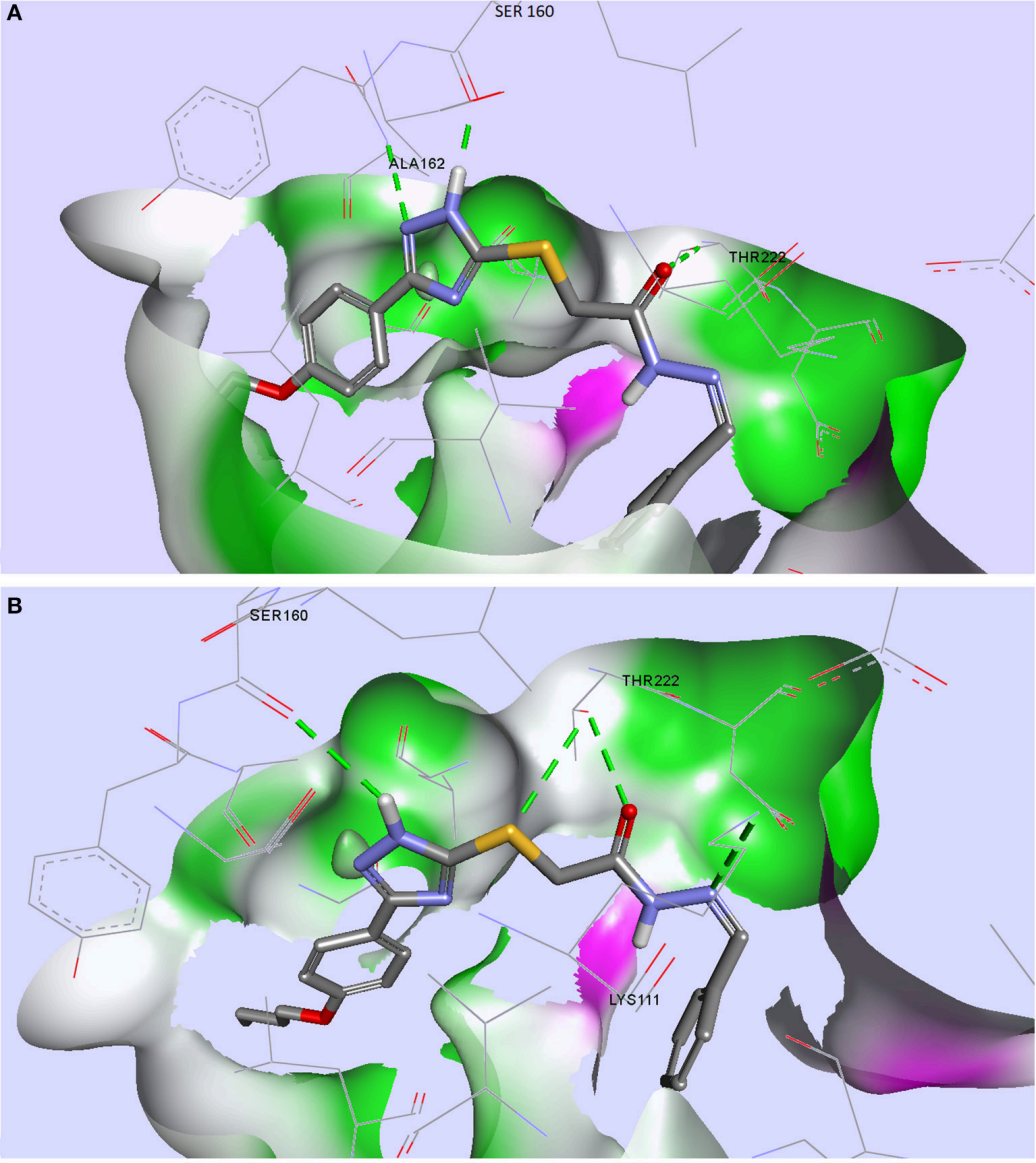
Compounds TZ53.7 **(A)** and TZ55.7 **(B)** docked in the active site of PDK1, PDB ID: 2PE1; HBs are depicted as green dotted lines.

From the compound selection docked in PDK1, two molecules, TZ53.7 and TZ55.7, showed adequate spatial orientation in the active site and superior binding characteristics compared to other analyzed structures. Of the two compounds, only TZ53.7 forms two key HBs with the hinge-region amino acids Ala162 and Ser160, respectively, similar to the reported indolinone-based PDK1 inhibitors and another hydrogen bridge with Thr222, which presumably would confer selectivity for the target protein (Islam et al., 2007) (Figure 5). On the other hand, TZ55.7 forms only one hinge-region interaction, a HB with Ser160, and another 3 interactions with amino acids Thr222 (2 HBs) and Lys211 (1 HB).

Compound TZ3a.7 was retained as a suitable inhibitor for VEGFR2; the binding pattern analysis of this compound was previously described in another study by our research group (Mioc et al., 2017b). This structure showed strong binding properties against VEGFR2. In this case the compound showed binding characteristics similar to other reported VEGFR2 back-pocket kinase inhibitors (Iwata et al., 2012), that do not interact with the hinge-region where the ATP's binding site resides. Furthermore, our model revealed that TZ3a.7 also interacts with the hinge-region as well, through multiple hydrophobic interactions (Mioc et al., 2017b).

### Chemistry

Synthesis of S-substituted 1*H*-3-R-5-mercapto-1,2,4-triazoles(4a-b, 5a, 6a-c) was carried out by an alkylation reaction of 3a-c with the respective alkyl halides [ethyl chloroacetate (i), chloroacetamide (ii), N-benzylideneamino-2-chloro-acetamide (iii)] in the presence of sodium ethoxide. Compounds 4a and 4b were obtained as intermediates in the synthesis of compounds 5a and 6a-c by a condensation reaction with ammonia for compound 6a and hydrazine hydrate followed by benzaldehyde for compounds 6b and 6c. This synthetic route proved to give low yields and it was problematic as well, in terms of compound purification in the case of compounds 6b and 6c, where two TLC spots with similar Rf values were noticed. Subsequently, we synthesized N-benzylideneamino-2-chloro-acetamide by using a method previously described in the literature (Suketaka and Narusawa, 1970) and used it as an alkylating agent for the synthesis of compounds 6a-c; similarly, we obtained chloroacetamide for the synthesis of compound 5a. These procedures gave higher yields and were less time consuming. Considering that over time, the evaluation of the biological activities of drug synthesis intermediates has revealed new highly active molecules, compounds 4a and 4b were tested for their biological activity as well, even though they were not part of the final docking selection.

### Biological evaluation

#### Cell viability

**Figure 6:**
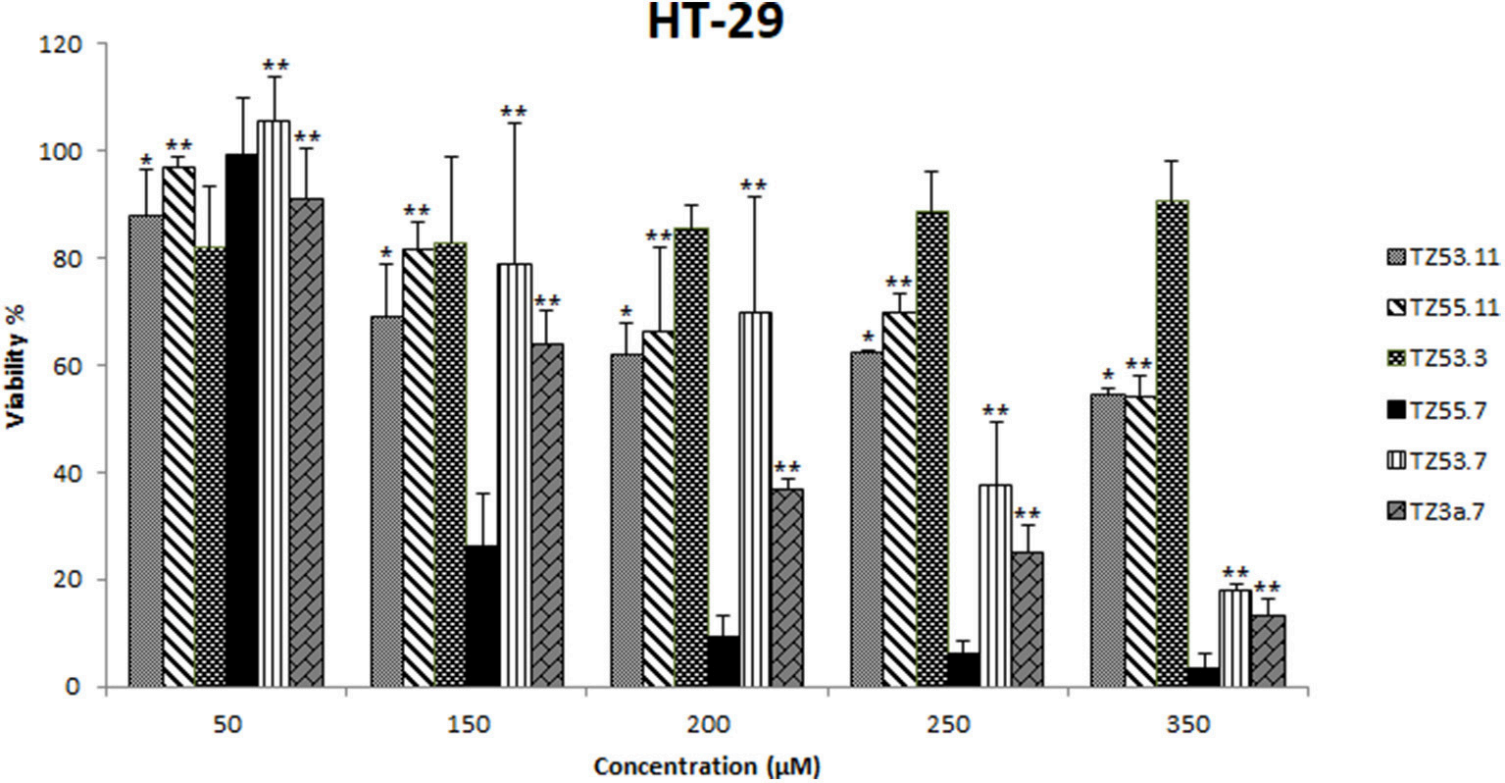
Cell viability recorded on HT-29 cell line, for the tested compounds TZ53.3, TZ53.7, TZ55.7, TZ3a.7, TZ53.11, TZ55.11, at concentrations of: 50, 150, 200, 250, and 350 μM; (*p < 0.05; **p < 0.01).

The cytotoxic effect of the six compounds (TZ53.3, TZ53.7, TZ55.7, TZ3a.7, TZ53.11, TZ55.11) was tested on the HT-29 colorectal cancer cell line by means of Alamar Blue assay that evaluates the metabolic function and cellular health; Alamar Blue is a redox indicator that provides multiple advantages over tetrazolium salts in terms of cell viability and metabolic assessment (Rampersad, 2012). Briefly, the active ingredient is the blue, water-soluble, non-toxic resazurin which is reduced in viable cells to the pink, highly fluorescent resorufin (Rampersad, 2012) that can be spectrophotometrically evaluated. Results are depicted in Figure 6.

HT-29 is a well differentiated colon cancer cell line documented among the fastest growing cultures (20-24 h doubling time), that shows the hyperactivating mutation of the PIK3CA oncogene and lacks the mutation of PTEN gene (tumor suppressor) which are two of the most altered genes in colorectal cancer (Ahmed et al., 2013); therefore HT-29 was chosen as target for the *in vitro* assays in the current study.

Most often, the development of new anticancer drug candidates is based on the assessment of the nanomolar *in vitro* potency assuming that the *in vivo* effect will follow the *in vitro* profile (Wong et al., 2012); according to this hypothesis compounds with lower IC_50_ values will have higher *in vivo* efficacy and lower toxicity. The calculated IC_50_ values are included in Table 2.

**Table 2:** IC_50_ (μM) values of the tested compounds TZ53.3, TZ53.7, TZ55.7, TZ3a.7, TZ53.11, TZ55.11, on the HT-29 cell line.

**Compound**	**HT-29 IC_50_ (μM)**
TZ53.11	437.84
TZ55.11	374.10
TZ53.3	–
TZ55.7	87.95
TZ53.7	239.25
TZ3a.7	149.25

One can notice that the compound with the most significant cytotoxic activity against HT-29 tumor cell line was found to be TZ55.7 (IC_50_ = 87.95 μM). According to the results obtained from molecular docking, TZ55.7 was retained as a candidate for the inhibition of the PDK1 enzyme. This enzyme plays an important role in the phosphorylation of a wide range of representatives of the AGC kinase family, of which the three most important are the three AKT isoforms (Angiolini et al., 2010; Medina, 2013), with well-defined roles in various types of cancer, including gastric and colorectal cancer (Kim et al., 2016), thus being a key node in the signaling transmission of the PI3K/AKT pathway. A major aspect is that, unlike PI3K or AKT, PDK1 exists in only one isoform coded by a single copy gene allowing the development of more selective and more efficient inhibitors (Hossen et al., 2015). PDK1 inhibitors act as antiproliferative agents in various forms of cancer, including colorectal cancer, as reported in the literature (Garcia-Echeveria and Sellers, 2008). Moreover, PDK1 inhibitors may cause the selective elimination of cancer stem cells responsible for tumor regeneration (Cunningham and Ruggero, 2013). The correlation between PDK1 inhibition and the antiproliferative effect on HT-29 cells was reported in a study of (Arico et al., 2002). In this study, the mechanism by which the COX-2 inhibitor, celecoxib, exerts its antiproliferative effect on HT-29 cells (with no COX-2 enzyme activity) has been investigated revealing a clear correlation with the indirect alteration of the PI3K/AKT signaling pathway by inhibiting the PDK1 enzyme (Arico et al., 2002). Taking into consideration all these data we can assume that the strong cytotoxic activity of TZ55.7 against HT-29 cells may be explained through the potential inhibition of PDK1; this assumption is supported by a patent issued by Merck in 2010 that describes the synthesis of several very strong PDK1 inhibitors with triazolic structure (Barile et al., 2012).

Compound TZ53.7 was also selected through molecular docking as PDK1 inhibitor but showed a relatively high IC_50_ value (239.25 μM); however, *in vitro* testing on cancer cells is based on the assumption that the cells retain their *in vivo* properties which is not always the case due to individual variations as well as culture adaptation and immortalization (Wong et al., 2012). Therefore further *in vivo* tests may be needed in order to completely eliminate this compound as potential anticancer agent or, on the contrary, validate it as such but acting in a dose-independent manner.

Compound TZ3a.7 was next ranked according to its cytotoxicity on the HT-29 tumor cell line. According to the molecular docking study, this compound obtained a very good score against VEGFR2, the main proangiogenic factor in various types of cancer (Araújo et al., 2015). In colon cancer, VEGFR2 regulates endothelial differentiation and its expression correlates with metastasis or tumor recurrence followed by poor prognosis (Liu et al., 2017). The activity results may therefore be attributed to VEGFR2 inhibition as the molecular docking indicates. In our previous work (Mioc et al., 2017b), the results of molecular docking were reported: compound TZ3a.7 formes two HBs with ASP1046 and two hydrophobic interactions with the Val898 and Leu1019 residues, respectively, located in the protein back pocket. Furthermore the phenyl ring of the compound hydrophobically interacts with the hinge region of the binding site (Mioc et al., 2017b). Previous papers also reported the antiproliferative activity of selective VEGFR2 inhibitors, such as GW654652 (indazolylpyrimidine derivative), against the HT-29 cell line (Dev et al., 2004). Compounds containing the 1,2,4-triazole moiety included in a 5-6 fused ring and possessing a nitrogen atom in position 1 were proven strong VEGFR2 inhibitors due to the HBs formed by N1-nitrogen with the -NH- group from Cys919 in the VEGFR2 structure (Oguro et al., 2013).

For compound TZ53.3, the determination of the IC_50_ value was not possible as opposed to other compounds that reduced cell viability in a dose-dependent manner. This type of cytotoxic activity was not recorded for TZ53.3; moreover, at higher concentrations, a decrease in the antiproliferative effect was noticed. According to the molecular docking results, compound TZ53.3 was selected as ERK2 inhibitor thus theoretically standing as a potential antitumor agent against colon cancer types with overexpressed signaling through the MAPK pathway. Fang *et al*. reported in 2015 that colon cancer development is directly related to the binding of ERK2 (a protein kinase which together with RAF and MEK forms the MAPK signaling pathway) to Leu163 and Val165 and phosphorylation of Ser148 in the structure of the overexpressed CSN6 (a subunit of COP9 signalosome), which correlates to poor prognosis (Fang et al., 2015). However the HT-29 colon cancer cell line exhibits the *BRAF*^V600^ mutation (Ahmed et al., 2013) that is present in about 10% of colorectal cancers and in most cases indicates the possibility of intrinsic/adaptive cell proliferation through the activation of other oncogenic signaling pathways therefore inducing resistance to MEK/ERK inhibition (Kirouac et al., 2017). Such a possibility is signaling through the PI3K/AKT pathway that was proven to mediate resistance against MAPK inhibition in *BRAF*^V600^–colorectal cancer where gene analysis revealed hyperactivated PI3K/AKT/mTOR signaling lacking any correlation with PIK3CA mutations (Kirouac et al., 2017). Collectively, the data suggest that in spite of the inhibitory activity of ERK2 induced by compound TZ53.3, the HT-29 colon cancer cell line may still continue to proliferate due to the *BRAF*^V600^ mutation, through the activation of alternative signaling pathways.

Thus, the interconnection that exists between various signaling pathways activated in colon cancer represents an obstacle difficult to bypass within the development of new targeted therapies with increased antitumor efficacy. A new strategy would consist in the simultaneous blocking of multiple signaling pathways; the double inhibition of various components belonging to the MAPK and PI3K signaling pathways led to clear superior outcomes in terms of colorectal cancer treatment compared to the single signaling pathway inhibition (Temraz et al., 2015).

The 6 compounds were also tested for cytotoxic effects against the normal cell line HaCaT (human keratinocytes) by using the Alamar assay and the same concentrations previously tested on the HT-29 colon cancer cell line. All compounds showed low antiproliferative activity against normal cells (cellular viability ranging between 85 and 97%) thus revealing high selectivity against the HT-29 tumor cell line. Collectively, by corroborating these data with our previous results (Mioc et al., 2017c) we may suggest that S-alkylated triazole derivatives exert stronger citotoxic activity against HT-29 cell line compared to their non-alkylated counterparts; however, they also revealed higher citotoxic effects against normal HaCaT cells. A second conclusion can be formulated in terms of the length of the alkyl chain grafted on the hydroxyl moiety in position 4′; it seems that in this case, compounds bearing a shorter alkyl chain (TZ53.11, TZ53.7) exhibited a stronger citotoxic activity when compared with the compounds containing a longer alkyl group (TZ57.11, TZ55.7). This could prove an important aspect for the future design of triazole derivatives, substituted with phenoxy alkyl radicals in the third position, as anticancer agents. Aliabadi et al. suggested in 2016 that the presence of -F or -NO_2_ as phenyl residue substituents in the molecule of 1,2,4-triazole derivatives led to increased citotoxic potency and selectivity against the HT-29 colorectal cancer cell line (Aliabadi et al., 2016); this hypothesis may generate future research directions for our synthetic derivatives.

Following these results, compounds recorded with the lowest IC_50_ (TZ53.7, TZ55.7, TZ3a.7) were subjected to additional biological evaluations, to verify if they inhibit the expression of their proposed protein targets (PDK1,VEGFR2), in the HT-29 tumor cell line.

#### Assessment of VEGFR2 concentration in culture medium

**Figure 7:**
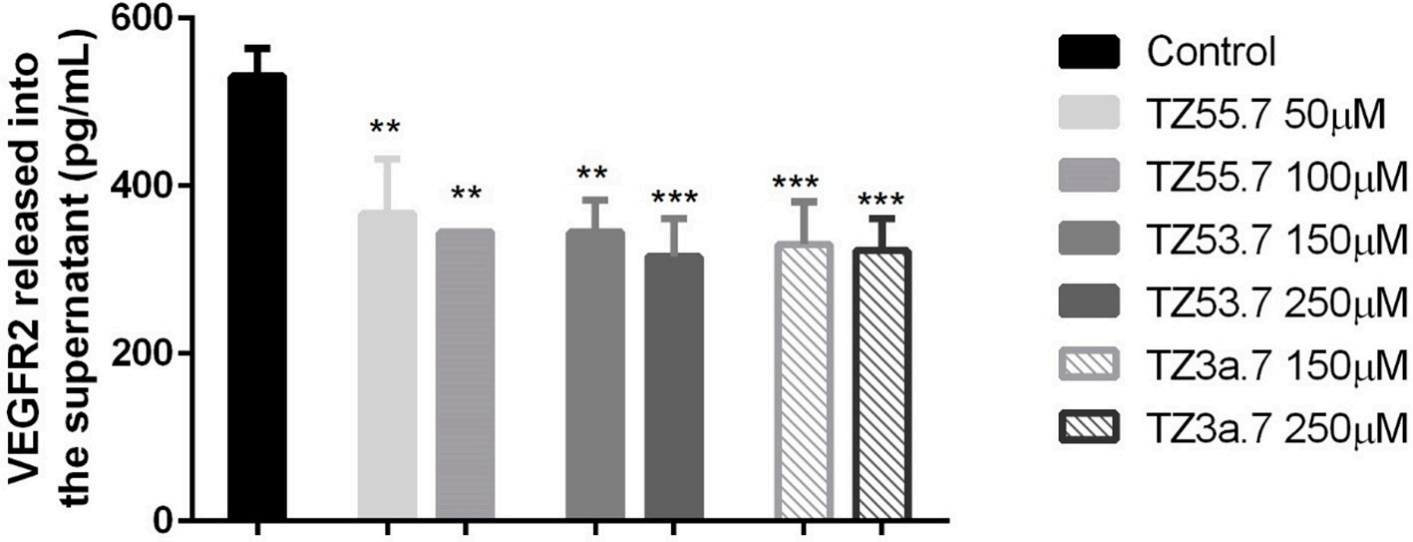
ELISA determination of VEGF-R2 secreted by HT-29 cells into culture medium after stimulation with test compounds (TZ55.7 - 50 and 100 μM, TZ53.7 - 150 and 250 μM) and TZ3a.7 - 150 and 250 μM) for 24 h. The values are expressed as mean ± standard deviation. The statistical significance was determined with One-way ANOVA using GraphPad Prism 6 software (**p < 0.01, ***p < 0.001).

To assess the concentration of VEGFR2 in the culture medium after stimulation with test compounds TZ55.7 (50 and 100 μM), TZ53.7 (150 and 250 μM) and TZ3a.7 (150 and 250 μM) an ELISA test was performed. The results indicated that the highest concentration of VEGFR2 was detected in the control samples (unstimulated cells). All test compounds induced an inhibitory effect on VEGFR2 expression as compared to control cells, but the most statistically significant inhibition was recorded for TZ3a.7 (150 and 250 μM), results that confirm the molecular docking analysis according to which TZ3a.7 is a suitable inhibitor for VEGFR2 (Figure 7).

Vascular endothelial growth factor receptor 2 (VEGFR2) is a type V receptor tyrosine kinase encoded by the *KDR* gene (Miettinen et al., 2012), known to be expressed mostly in endothelial cells (Liu et al., 2017). This receptor after being activated, by responding to VEGF-A signal, triggers a phosphorylation cascade, promoting regulation of nuclear targets and increased endothelial proliferation and migration (Miettinen et al., 2012). Expression of VEGFR2 protein was also detected in different tumor cells, like: human colorectal, breast and non-small cell lung cancers (Holzer et al., 2013), hemangiomas, angiosarcomas, malignant melanoma and diffuse large B-cell lymphoma (Miettinen et al., 2012). It was also proved that, in colorectal cancer cells, activation of VEGF/VEGFR2 signaling initiates STAT3 phosphorylation and promotes tumor development (Mager et al., 2016). Besides the role in angiogenesis, overexpression of VEGFR2 was associated with invasion, metastasis and poor prognosis in different cancers. In addition, it also regulates the senescence, apoptosis and proliferation of tumor cells (Holzer et al., 2013; Foersch et al., 2015). Based on these data, VEGFR2 is considered a valid target for anti-cancer treatment. In the present study it was shown that TZ3a.7 presents a specificity for VEGFR2 binding, results that were confirmed by ELISA test. It was also observed that the decreased expression of VEGFR2 in the culture medium was also observed, not only for TZ3a.7, but also for the other two tested compounds, TZ55.7 and TZ53.7 that were described as inhibitors of PDK1. These findings could be explained by the fact that PDK1 overexpression augments VEGF-A—induced cell migration, whereas PDK1 knockout, completely suppressed migration capacity of embryoid bodies-derived endothelial cells (Di Blasio et al., 2017).

#### Immunofluorescence staining

**Figure 8:**
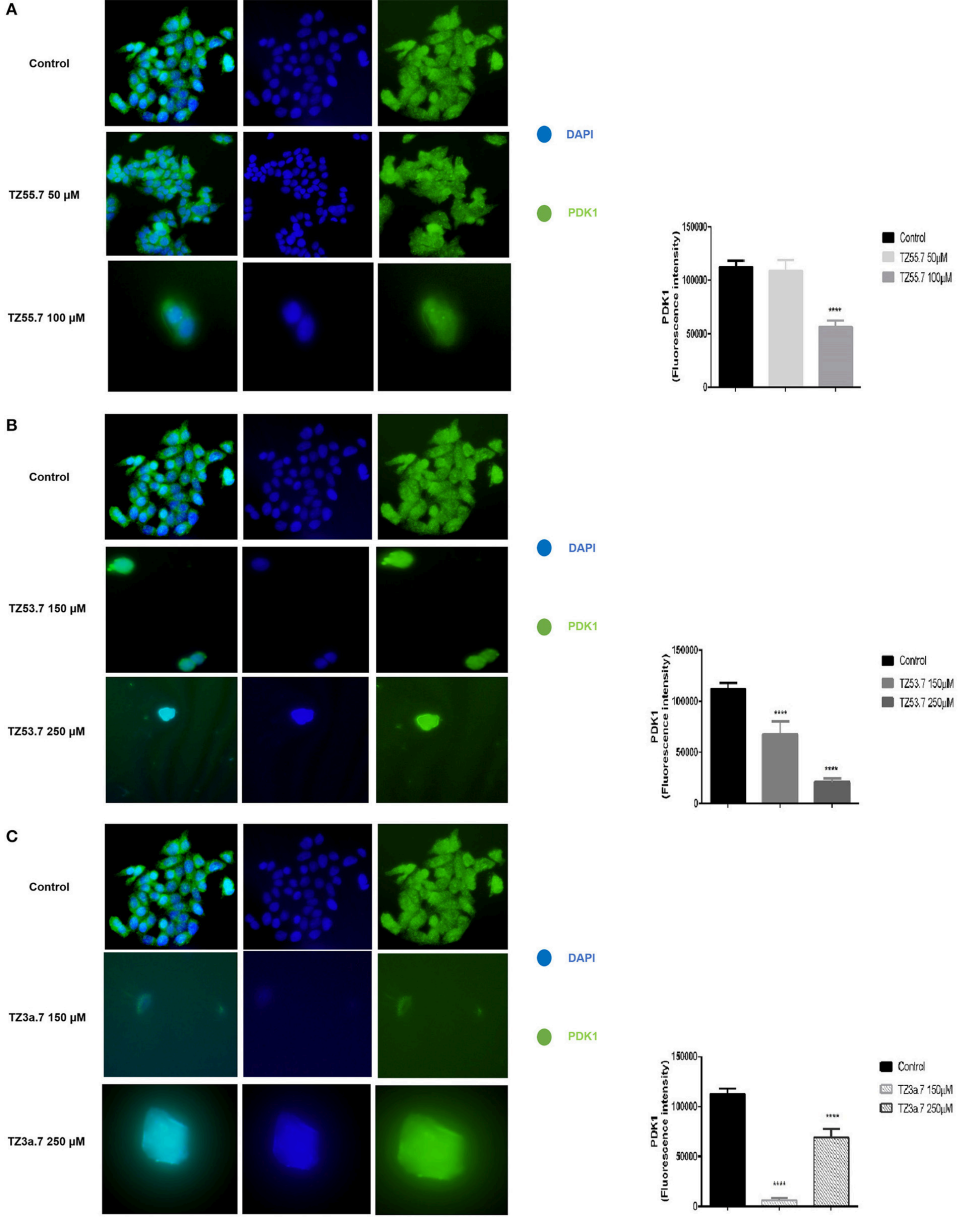
Immunostaining of PDK1 expression in HT-29 cells stimulated with TZ55.7 (50 and 100 μM) **(A)**, TZ53.7 (150 and 250 μM) **(B)**, TZ3a.7 (150 and 250 μM) **(C)**, for 24 h compared to control cells (unstimulated cells). Quantification of the fluorescence intensity was assessed for five randomly selected areas using ImageJ software and is presented in graphical representation. The statistical significance was performed with One-way ANOVA using GraphPad Prism 6 software (****p < 0.0001).

Immunofluorescence staining was performed for PDK1 in HT-29 cells to confirm the data presented in the molecular docking results that TZ55.7 and TZ53.7 are inhibitors of PDK1 expression. All cells treated with test compounds stained positive to PDK1 (Figure 8). As shown in Figure 8A the cells stimulated with TZ55.7 – 50 μM did not present modifications of cell morphology, their features in terms of shape, number and adherent capacity being similar with the ones of control cells, results that confirm the cell viability experiments. TZ55.7 (50 μM) solution did not affect the cells' nucleus (DAPI – blue staining). In the case of TZ55.7 – 100 μM, a fade staining of both DAPI and PDK1, can be observed, which indicates a cytotoxicity induced by the test compound. The quantification of the fluorescence intensity indicated that TZ55.7 at 50 μM reduced the expression of PDK1 but the decrease was not statistically significant, whereas at 100 μM the reduction was significant, which proves the inhibitory effect of TZ55.7 on PDK1 expression. TZ53.7 (150 and 250 μM) induced changes in cells shapes as compared to control cells; the stimulated cells became round and at the highest concentrations, the nucleus shape was also modified, these aspects being specific signs for cytotoxicity and apoptosis. Quantification of fluorescence staining revealed a potent inhibitory activity of TZ53.7 on PDK1 expression, the observed effect being dose-dependent (Figure 8B). In the case of TZ3a.7 (150 and 250 μM), which is a specific inhibitor for VEGFR2, a significant decrease of staining at the lowest concentration−150 μM was also observed, whereas at 250 μM, the recorded effect was less intense (Figure 8C).

#### Cell cycle analysis and annexin V apoptosis assay

**Figure 9:**
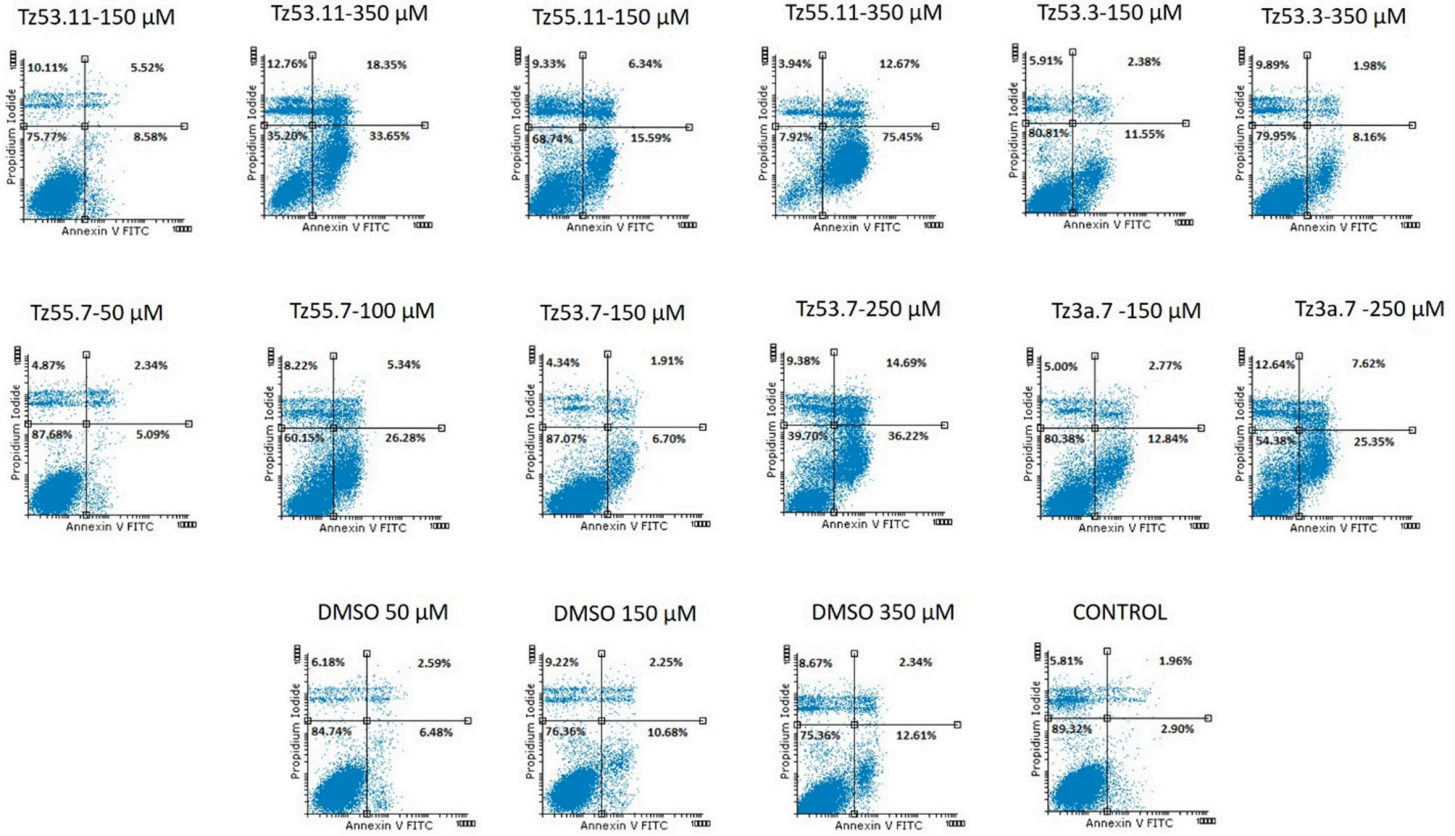
Representative dotplots for the flow cytometric analysis of HT-29 cell apoptosis.

Cancer survival indicates the prevalence of cell proliferation at the expense of apoptosis (Kapral et al., 2017); therefore, pro-apoptotic drugs exert an antitumor effect and may represent a research direction toward cancer treatment. We investigated the six triazole compounds in terms of apoptosis triggering in order to identify their potential antitumor mechanism. Figure 9 shows representative dotplots for the flow cytometric analysis of HT-29 cell apoptosis while Table 3 exhibits the mean values ± standard deviation of three separate experiments.

**Table 3:** Viability of HT-29 human colorectal adenocarcinoma cell line using Annexin V/PI analysis.

**Compound-concentration**	**Viable cells (%)**	**Early apoptotic cells (%)**	**Late apoptotic cells (%)**	**Necrotic cells (%)**
TZ53.11-150 μM	75.78 ± 11.38	8.59 ± 4.07	5.52 ± 3.14	10.11 ± 4.18
TZ53.11-350 μM	35.21 ± 0.23	33.66 ± 0.29	18.35 ± 0.20	12.77 ± 0.27
TZ55.11-150 μM	68.74 ± 15.84	15.59 ± 9.53	6.34 ± 3.40	9.33 ± 2.94
TZ55.11-350 μM	7.93 ± 0.60	75.46 ± 0.75	12.68 ± 0.01	3.94 ± 0.18
TZ53.3-150 μM	80.82 ± 9.90	11.55 ± 6.91	2.38 ± 0.86	5.92 ± 1.47
TZ53.3-350 μM	79.96 ± 7.26	8.16 ± 4.51	1.98 ± 0.38	9.90 ± 2.44
TZ55.7-50 μM	87.68 ± 2.54	5.10 ± 2.24	2.35 ± 0.25	4.87 ± 0.30
TZ55.7-100 μM	60.16 ± 1.18	26.28 ± 1.12	5.35 ± 0.02	8.22 ± 0.04
TZ53.7-150 μM	87.08 ± 1.63	6.67 ± 2.48	1.92 ± 0.10	4.34 ± 0.86
TZ53.7-250 μM	39.70 ± 2.04	36.22 ± 0.93	14.69 ± 1.16	9.38 ± 0.04
TZ3a.7-150 μM	80.38 ± 6.40	12.84 ± 7.36	2.77 ± 0.38	5.00 ± 0.59
TZ3a.7-250 μM	54.38 ± 17.97	25.35 ± 11.41	7.62 ± 1.51	12.64 ± 5.10
DMSO-50 μM	84.74 ± 3.08	6.48 ± 1.04	2.60 ± 0.64	6.18 ± 1.40
DMSO-150 μM	76.36 ± 11.55	10.69 ± 5.41	2.35 ± 0.77	8.67 ± 3.91
DMSO-350 μM	77.83 ± 11.12	12.62 ± 6.87	2.25 ± 1.06	9.23 ± 4.65
Control	89.32 ± 3.10	2.90 ± 0.57	1.97 ± 0.77	5.81 ± 2.35

One can notice (Figure 9) that all compounds exerted an apoptotic effect in the HT-29 cancer cells, enhancing the percentage of early apoptotic and late apoptotic cells as follows: for the lowest concentration of each compound, the recorded apoptotic effect was due to the solvent (dimethylsulfoxide, DMSO) which increased the percentage of early apoptotic cells from 2.90% ± 0.57 (untreated cells) up to 10.69% ± 5.41. However, the higher concentrations of all tested compounds produced an increased percentage of both early and late apoptotic cells, clearly superior to the effect produced by the solvent (12.62% ± 6.87—percentage of early apoptotic cells). The percentage of the early apoptotic cells after treatment with the tested compounds increased up to 75.46% ± 0.75 (for TZ55.11 which exerted the strongest apoptotic effect). These observations lead to the conclusion that the apoptotic effect appears only at higher doses.

**Figure 10:**
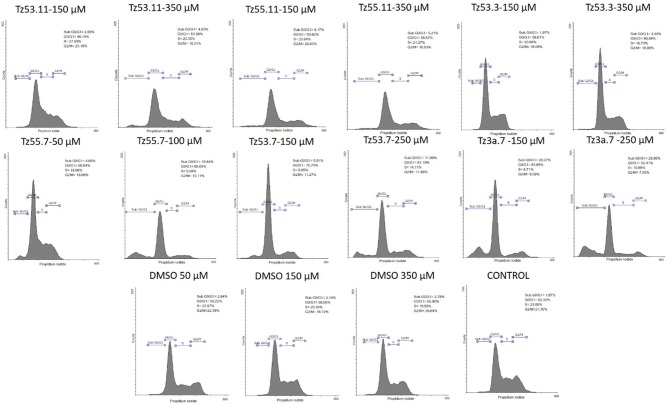
Representative histograms for the flow cytometric analysis of HT-29 cell cycle stimulated with test compounds.

Representative histograms for the flow cytometric analysis of HT-29 cell cycle are presented in Figure 10 while Table 4 displays the average values ± standard deviation of three separate experiments.

**Table 4:** Viability of HT-29 human colorectal adenocarcinoma cell line induced by the tested compounds in the four cell cycle phases; average values ± standard deviation of three separate experiments.

**Compound**	**Sub G0/G1 (%)**	**G0/G1 (%)**	**S (%)**	**G2/M (%)**
TZ53.11-150 μM	2.05 ± 0.03	46.14 ± 6.08	27.59 ± 0.97	23.16 ± 6.11
TZ53.11-350 μM	4.63 ± 1.64	53.08 ± 6.96	23.35 ± 2.72	18.31 ± 6.36
TZ55.11-150 μM	4.17 ± 1.81	50.92 ± 4.25	23.94 ± 0.02	20.83 ± 2.53
TZ55.11-350 μM	5.21 ± 0.07	55.57 ± 4.55	21.27 ± 3.02	16.63 ± 2.74
TZ53.3-150 μM	1.97 ± 1.10	59.01 ± 0.73	20.68 ± 2.33	18.06 ± 2.44
TZ53.3-350 μM	3.44 ± 1.79	60.58 ± 2.11	16.73 ± 1.14	18.96 ± 0.73
TZ55.7-50 μM	4.66 ± 0.90	56.64 ± 4.26	18.08 ± 1.70	19.89 ± 1.36
TZ55.7-100 μM	19.44 ± 5.64	65.00 ± 7.40	5.08 ± 6.41	10.11 ± 6.70
TZ53.7-150 μM	5.91 ± 0.35	75.74 ± 6.89	6.85 ± 4.68	11.27 ± 2.63
TZ53.7-250 μM	11.89 ± 3.27	61.19 ± 8.83	14.11 ± 8.85	11.89 ± 2.88
TZ3a.7-150 μM	20.27 ± 7.02	63.65 ± 7.02	6.71 ± 7.67	8.59 ± 3.80
TZ3a.7-250 μM	28.86 ± 9.49	52.41 ± 9.49	10.99 ± 5.63	7.55 ± 3.77
DMSO 50 μM	2.64 ± 0.42	50.22 ± 1.74	23.97 ± 0.89	22.38 ± 0.84
DMSO 150 μM	2.14 ± 0.61	56.05 ± 0.78	25.35 ± 0.11	16.12 ± 1.06
DMSO 350 μM	2.78 ± 0.27	55.90 ± 0.89	19.59 ± 1.32	20.64 ± 0.99
Control	1.97 ± 0.64	52.32 ± 0.64	23.00 ± 0.64	21.30 ± 1.47

The result of treating HT-29 cells with TZ53.11, TZ55.11, and TZ53.3 was a slight cell arrest in the sub G0/G1 phase (apoptotic cells). Also, for compound TZ53.3 a slight increase in the percentage of cells in G0/G1 phase was noticed. However, cell treatment with TZ55.7, TZ53.7, and TZ3a.7, respectively, led to a significant cell cycle arrest in both sub G0/G1 and G0/G1 phase thus indicating a strong apoptotic effect.

The apoptotic activity of PI3K/AKT/mTOR signaling pathway modulators was also tested in previous studies; as an example, small molecule PDK1 inhibitors revealed clear pro-apoptotic effects on the MDA-468 breast cancer cells while no apoptosis was noticed on the HCT-116 colon cancer cell line (Feldman et al., 2005). Another study reported the inhibition of AKT/mTOR pathway by inositol hexaphosphate presumably due to PI3K inhibition thus reducing proliferation and inducing apoptosis in colon cancer cells (Kapral et al., 2017). 1,2,4-triazole derivatives were identified as selective cytotoxic agents against tumor cells (K-562, A549 and PC-3) by activating the extrinsic and intrinsic apoptosis pathways (Kulabas et al., 2016). Our results concerning the apoptotic activity of the three compounds—TZ55.7, TZ53.7, and TZ3a.7—that also exhibited the lowest IC_50_ values are consistent with previously published data and suggest the hypothesis that their antitumor activity is due to apoptosis induction. However, compounds such as TZ55.11, despite its high IC_50_ value, was found to exert strong apoptotic effect. Taken together, these data suggest that the antitumor activity of the three most active compounds—TZ55.7, TZ53.7, and TZ3a.7—has a pro-apoptotic component but may simultaneously involve additional mechanisms that may become the object of future studies.

## Conclusions

This study reported the synthesis of a novel series of S-substituted 1*H*-3-R-5-mercapto-1,2,4-triazoles derivatives, selected by docking-based virtual screening and their biological evaluation as antiproliferative agents against the HT-29 colorectal cancer cell line. Our results showed that S-alkylated triazole derivatives exert strong citotoxic activity against the HT-29 cell line. In terms of docking studies we noticed that the S-substituted side chain that contained a –CO-NH-N = C– group gave higher scores to the respective compounds. Also the length of the alkyl chain grafted on the hydroxyl moiety in position 4′ seemed to influence the antiproliferative activity. In this case, compounds bearing a shorter alkyl chain (TZ53.11, TZ53.7) exhibited a stronger citotoxic activity when compared with the compounds containing a longer alkyl group (TZ57.11, TZ55.7). Compound TZ55.7, which was retained as a possible PDK1 inhibitor, exhibited the most significant cytotoxic activity against the HT-29 tumor cell line (IC_50_ = 87.95 μM). The same compound alongside compound TZ53.7, decreased PDK1 expression, in the HT-29 tumor cell line, in a dose dependent manner. Also TZ3a.7 was shown to reduce VEGFR2 expression in the HT-29 tumor cell line. Compounds TZ55.7, TZ53.7, and TZ3a.7 induced cell cycle arrest in both subG0/G1 and G0/G1 phase. Given its IC50 value and apoptotic activity, compound TZ55.7 could prove an important scaffold for future structural design in developing highly efficient antiproliferative agents.

## Author contributions

MM and VB were responsible for the synthesis and purification of the studied compounds. Compound physico-chemical characterization were done by RG, MB-P, and VB. The docking study was conducted by MM, SA, and LK. The biological studies from this work were conducted by CO, CT, AM, DC, and CD. CS has elaborated the final version of the manuscript, corrected the language and critically revised/evaluated the scientific work.

### Conflict of interest statement

The authors declare that the research was conducted in the absence of any commercial or financial relationships that could be construed as a potential conflict of interest.
