# Supplementary material for: Design, Synthesis and Biological Activity Evaluation of S-Substituted 1H-5-Mercapto-1,2,4-Triazole Derivatives as Antiproliferative Agents in Colorectal Cancer
Source: Front Chem. 2018 Aug 23;6:373. doi: 10.3389/fchem.2018.00373 (PMC6134806; doi:10.3389/fchem.2018.00373)
Supplement: Supplementary file 1 [file Data_Sheet_1.DOCX]

# SUPPLEMENTARY MATERIAL

**FT-IR, MNR SPECTRA OF SUBSTITUTED 1H-5-MERCAPTO-1,2,4-TRIAZOLE DERIVATIVES**

**
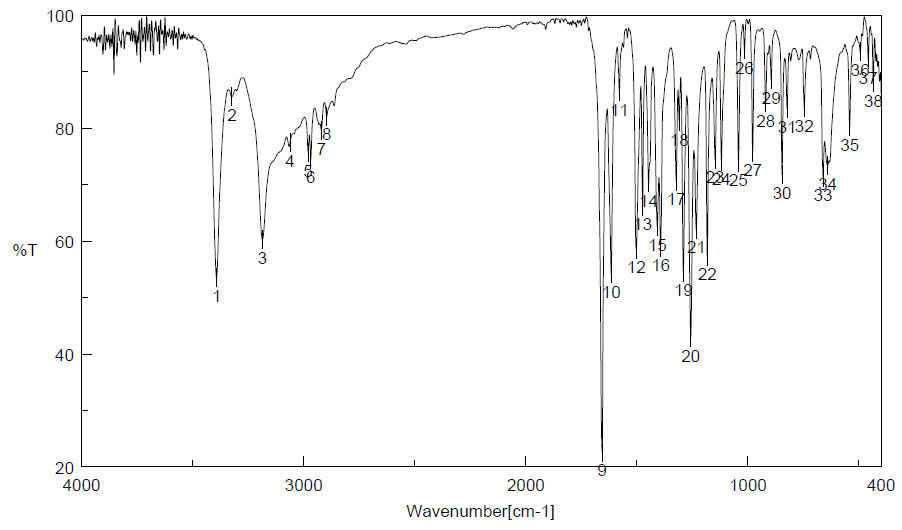

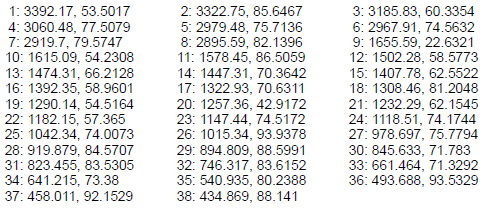
**

Figure 1.FTIR spectra of compound 1*H*-3-(4-ethoxyphenyl)-5-aminocarbonyl-methylsulfanyl-1,2,4-triazole (TZ53.3)

**

**Figure 2. 1H-MNR spectra of compound 1*H*-3-(4-ethoxyphenyl)-5-aminocarbonyl-methylsulfanyl-1,2,4-triazole (TZ53.3)

**
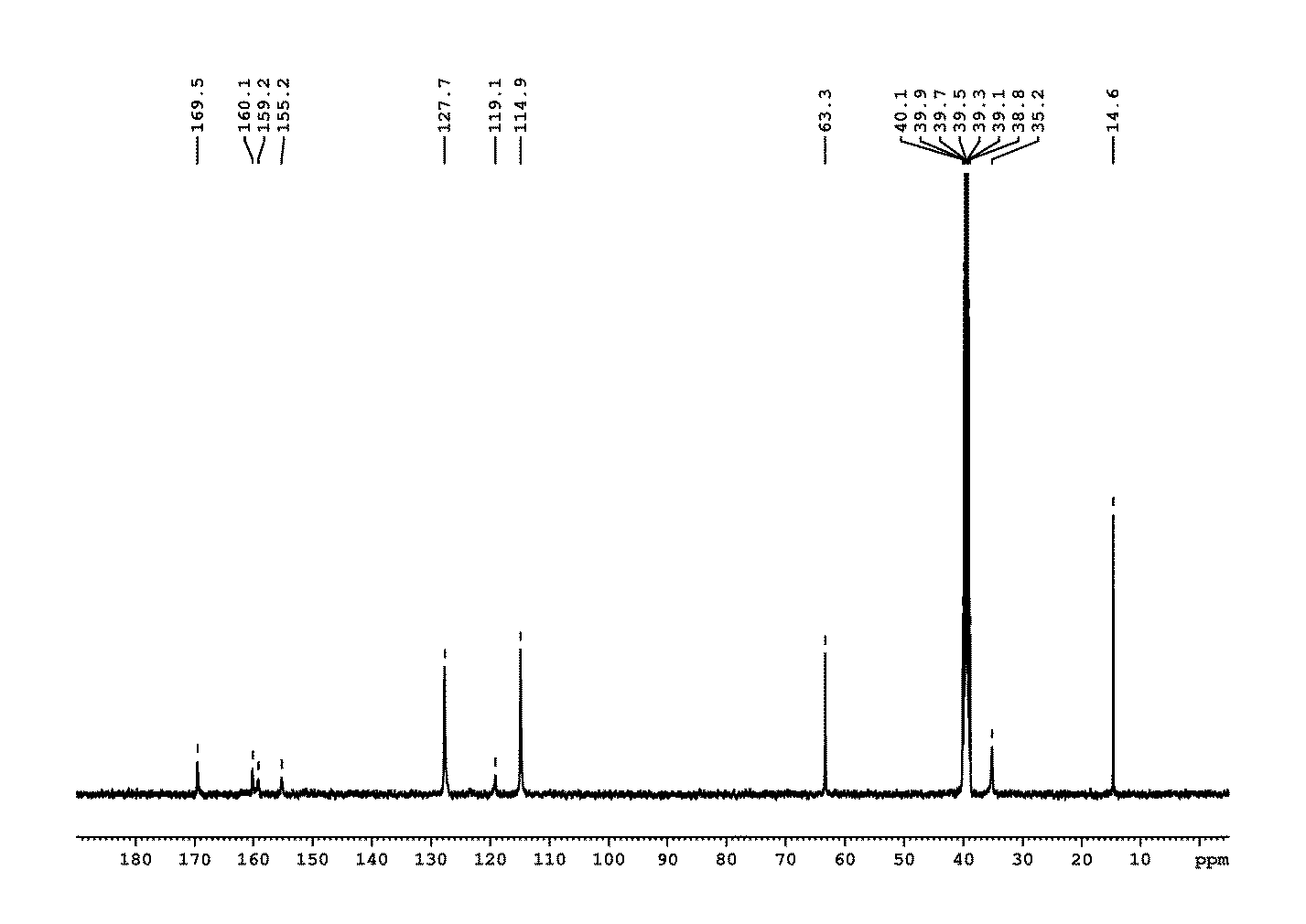
**Figure 3. 13C-MNR spectra of compound 1*H*-3-(4-ethoxyphenyl)-5-aminocarbonyl-methylsulfanyl-1,2,4-triazole (TZ53.3)

**
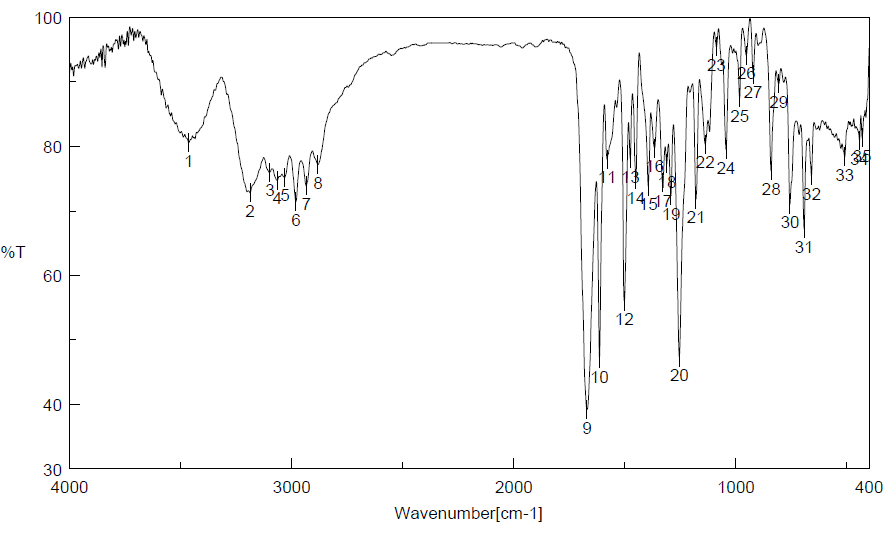

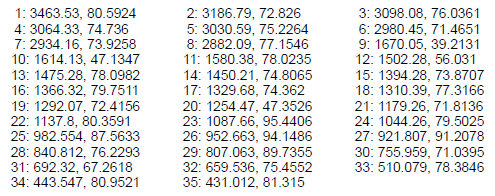
**

Figure 4.FTIR spectra of compound1*H*-3-(4-ethoxyphenyl)-5-benzylidenehydrazino-carbonyl-methylsulfanyl-1,2,4-triazole (TZ53.7)

**
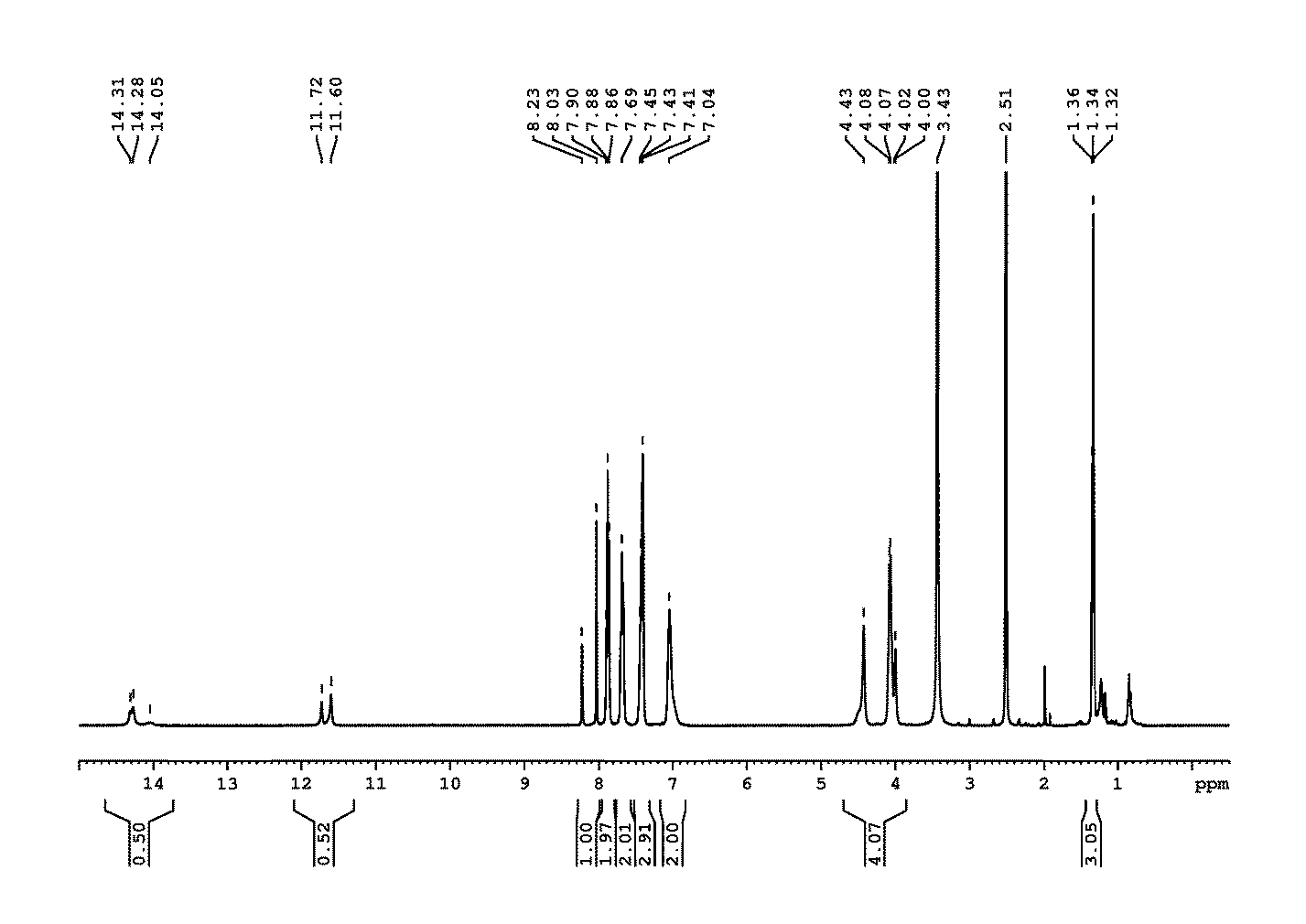
**Figure 5. 1H-MNR spectra of compound1*H*-3-(4-ethoxyphenyl)-5-benzylidenehydrazino-carbonyl-methylsulfanyl-1,2,4-triazole (TZ53.7)

**
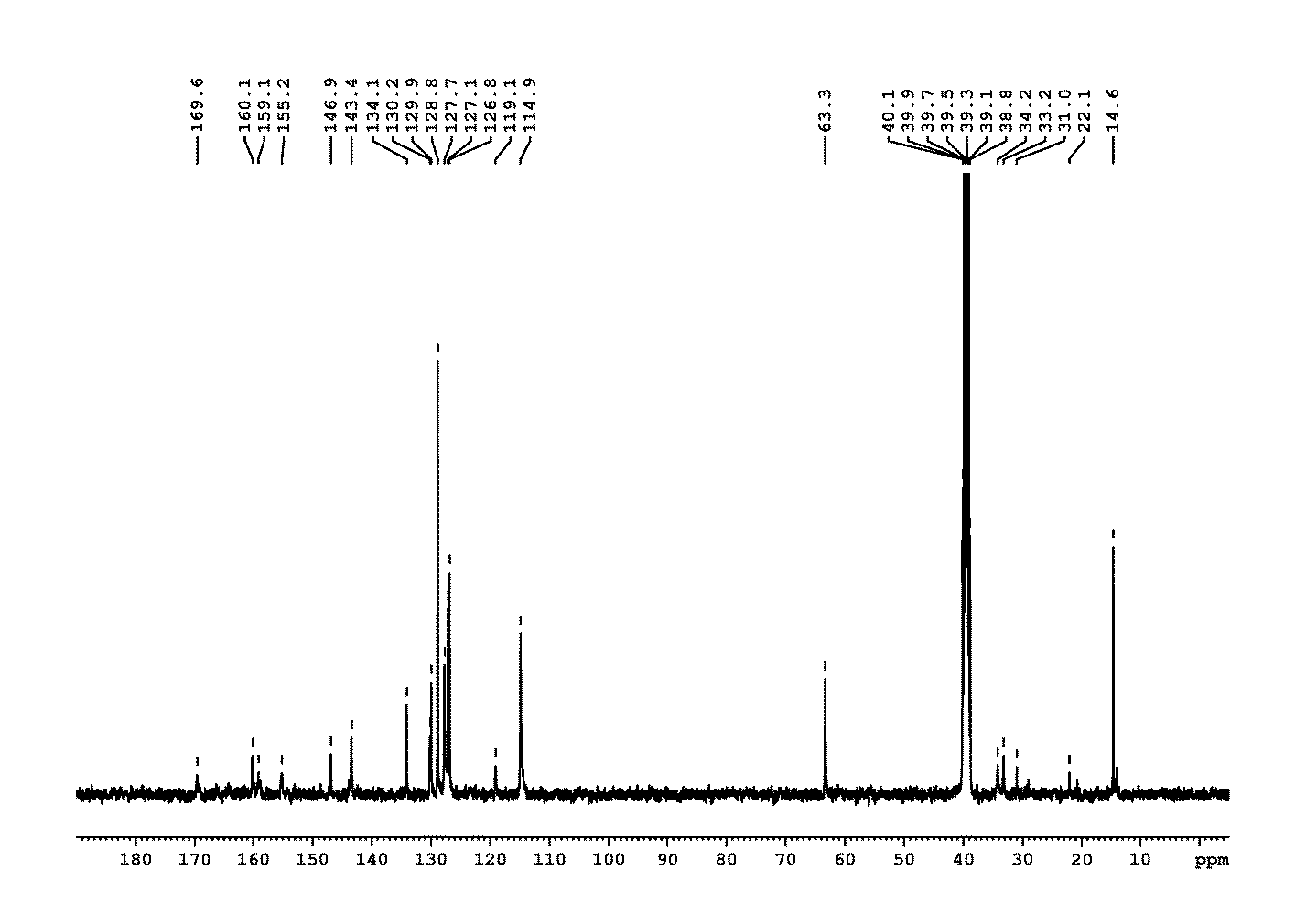
**Figure 6. 13C-MNR spectra of compound1*H*-3-(4-ethoxyphenyl)-5-benzylidenehydrazino-carbonyl-methylsulfanyl-1,2,4-triazole (TZ53.7)

**
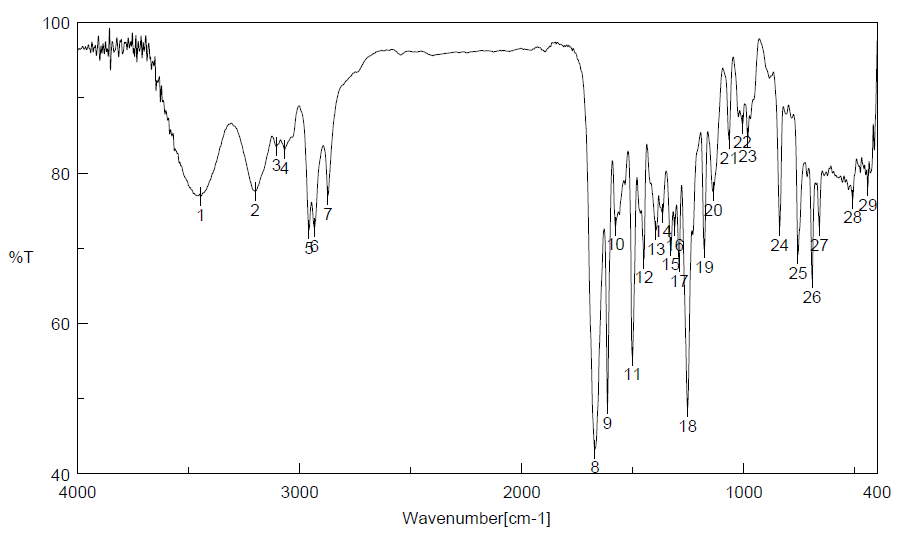

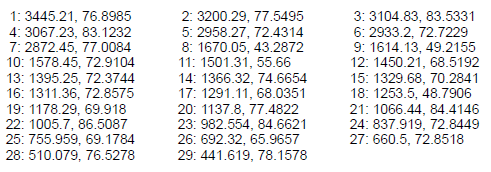
**

Figure 7. FTIR spectra of compound1*H*-3-(4-n-butoxyphenyl)-5-benzylidenehydrazino-carbonyl-methylsulfanyl-1,2,4-triazole (TZ55.7)

**
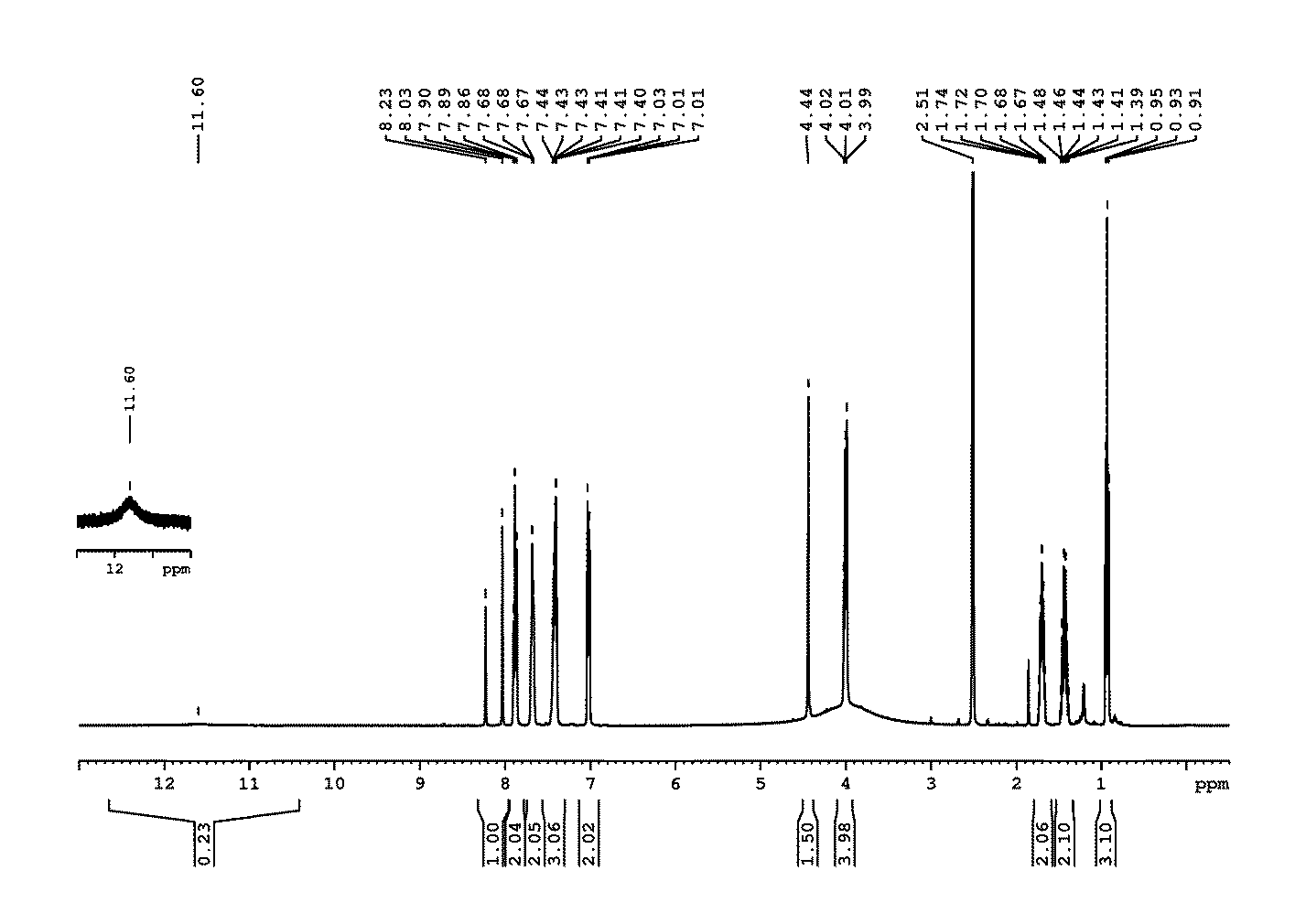
**Figure 8. 1H-MNR spectra of compound1*H*-3-(4-n-butoxyphenyl)-5-benzylidenehydrazino-carbonyl-methylsulfanyl-1,2,4-triazole (TZ55.7)

**
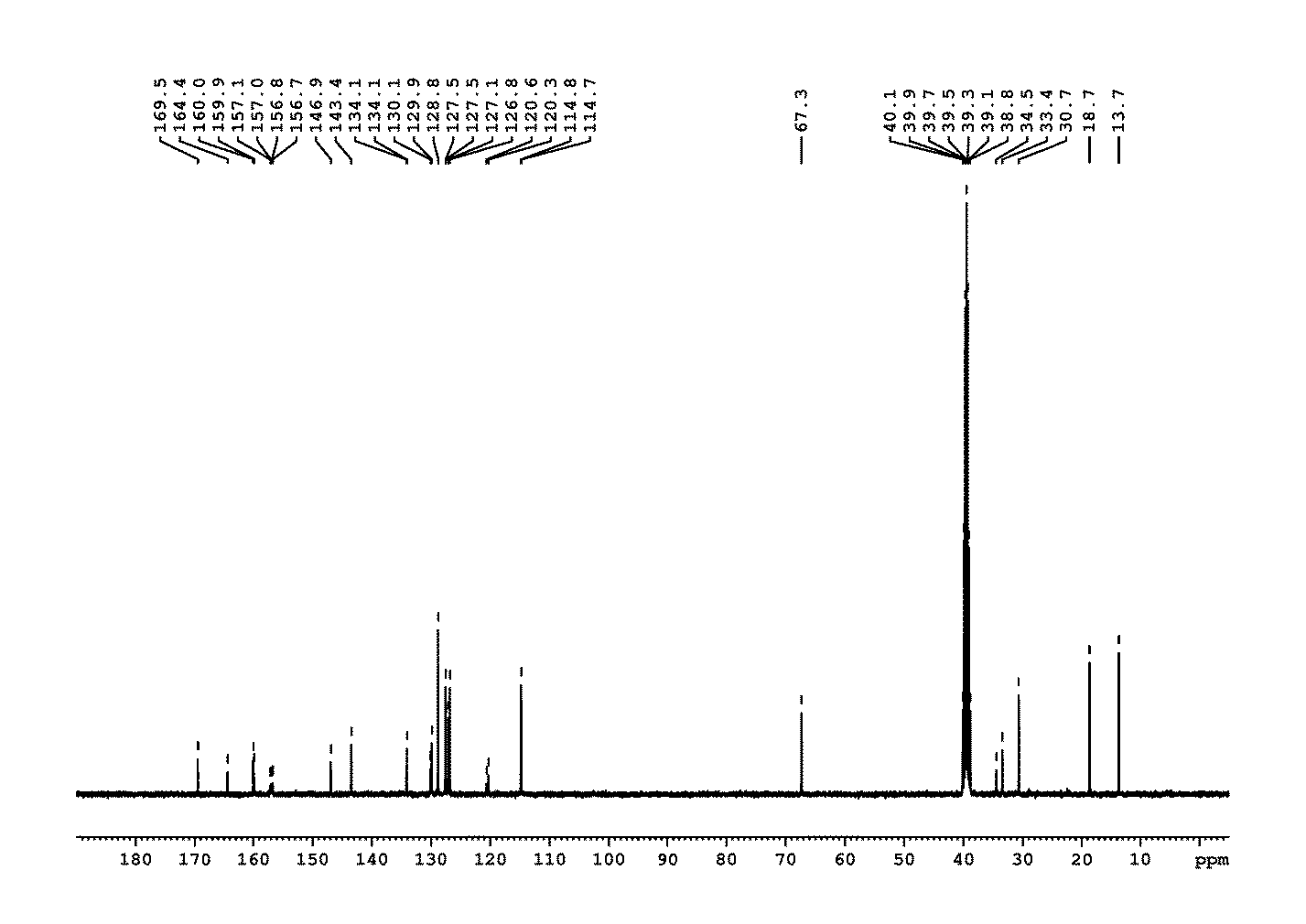
**Figure 9. 13C-MNR spectra of compound1*H*-3-(4-n-butoxyphenyl)-5-benzylidenehydrazino-carbonyl-methylsulfanyl-1,2,4-triazole (TZ55.7)

**
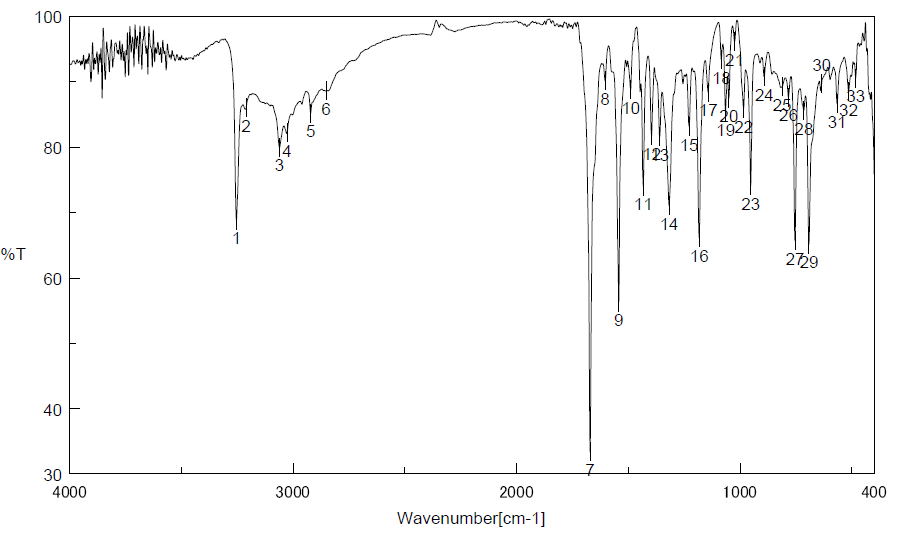

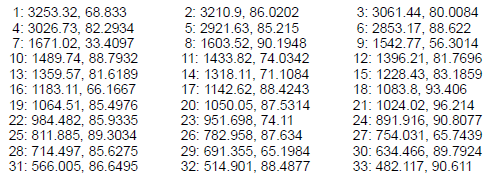
**

Figure 10. FTIR spectra of compound1*H*-3-stiril-5-benzylidenehydrazino-carbonyl-methylsulfanyl-1,2,4-triazole (TZ3a.7)

**
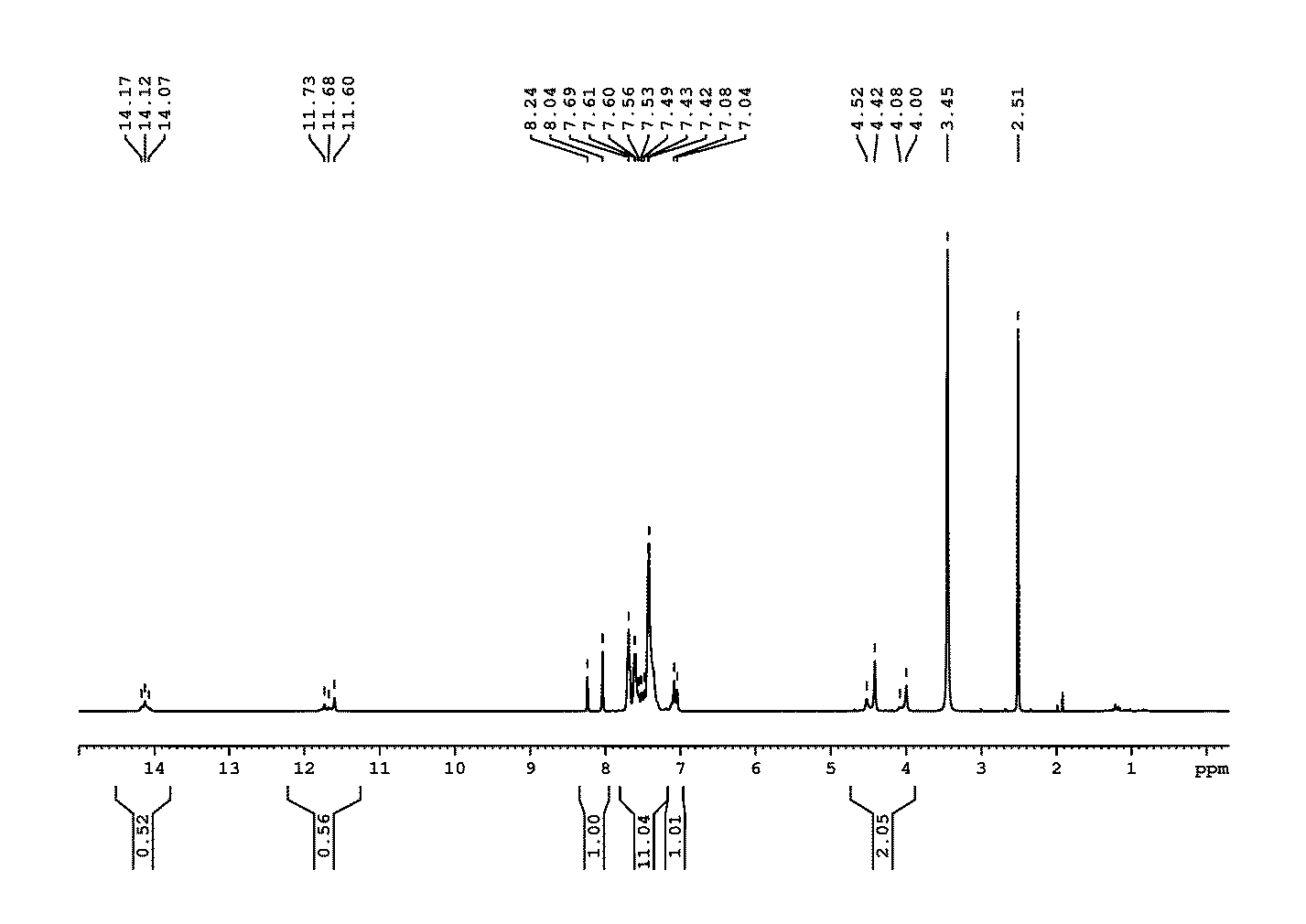
**Figure 11. 1H-MNR spectra of compound1*H*-3-stiril-5-benzylidenehydrazino-carbonyl-methylsulfanyl-1,2,4-triazole (TZ3a.7)

**
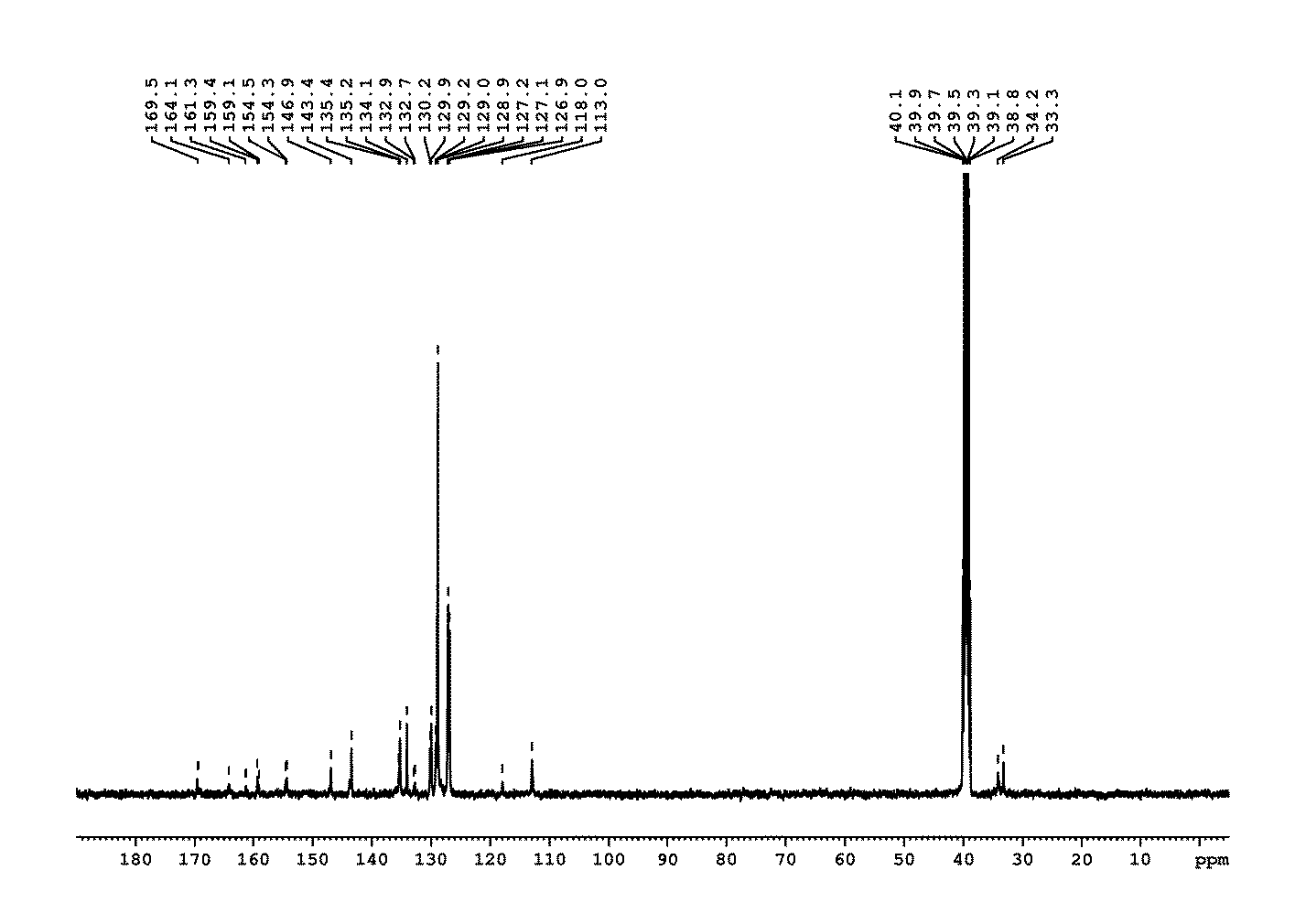
**Figure 12. 13C-MNR spectra of compound1*H*-3-stiril-5-benzylidenehydrazino-carbonyl-methylsulfanyl-1,2,4-triazole (TZ3a.7)

**
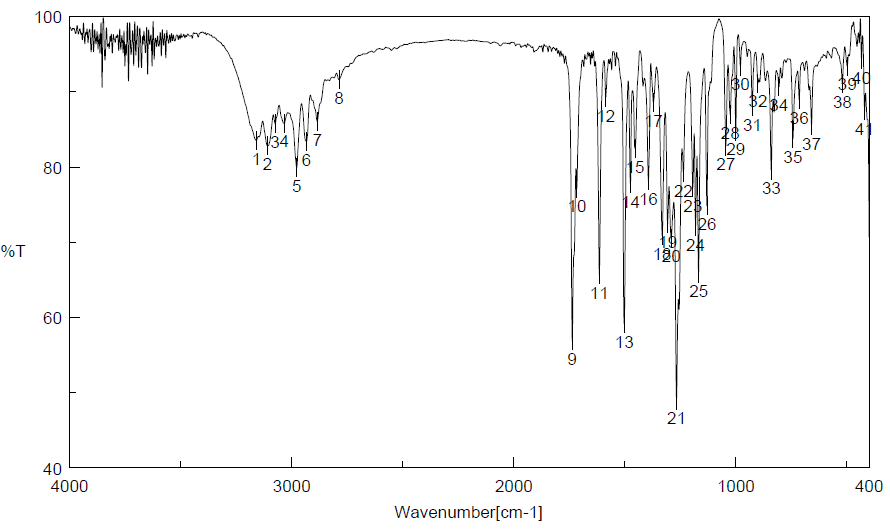

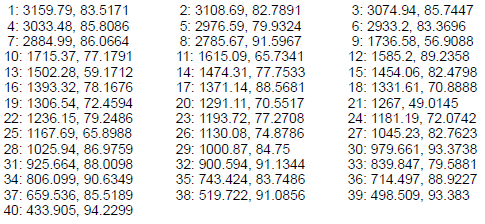
**

Figure 13. FTIR spectra of compound1*H*-3-(4-ethoxyphenyl)-5-ethoxycarbonyl-methylsulfanyl-1,2,4-triazole (TZ 53.11)

**
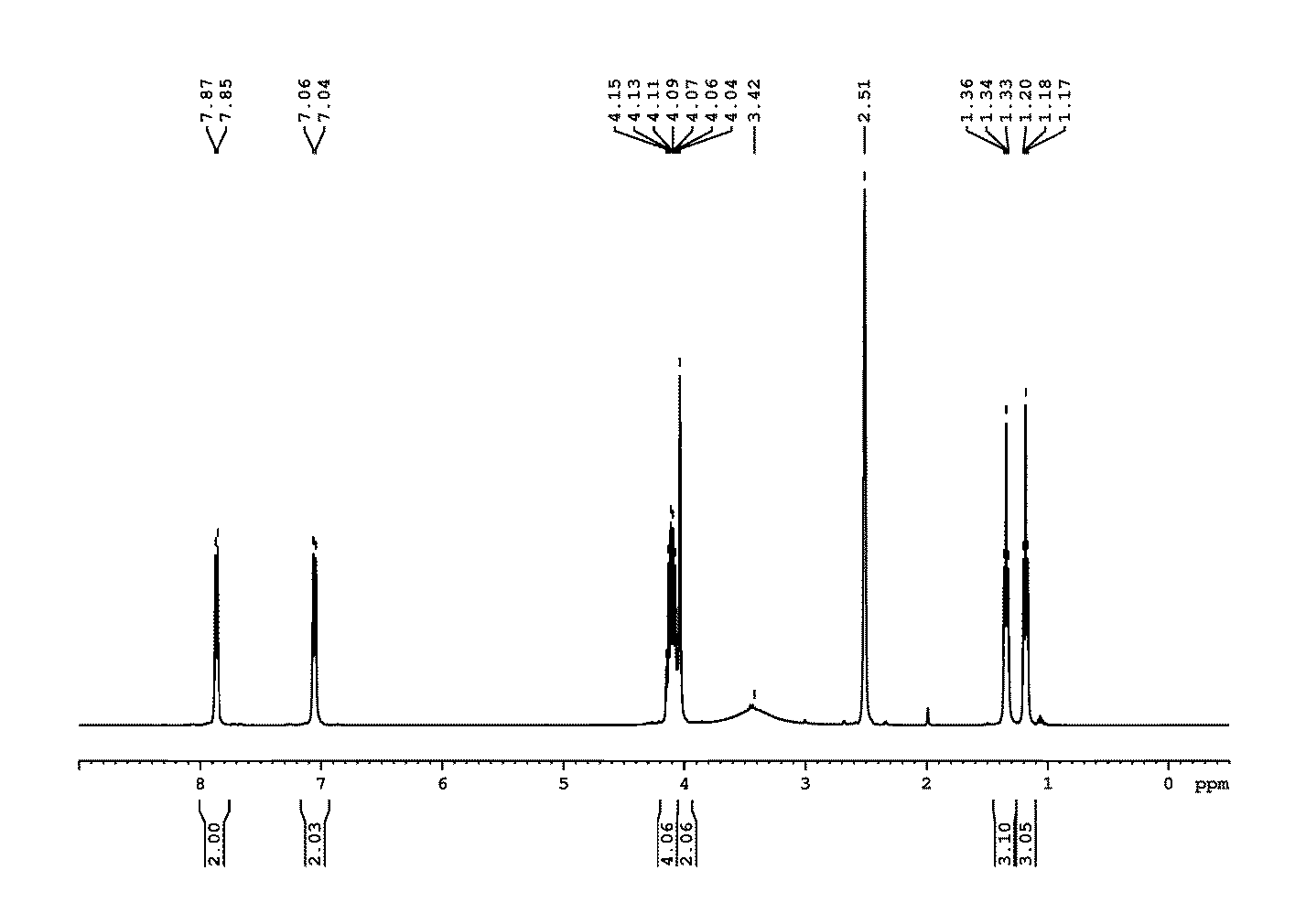
**Figure 14. 1H-MNR spectra of compound1*H*-3-(4-ethoxyphenyl)-5-ethoxycarbonyl-methylsulfanyl-1,2,4-triazole (TZ 53.11)

**
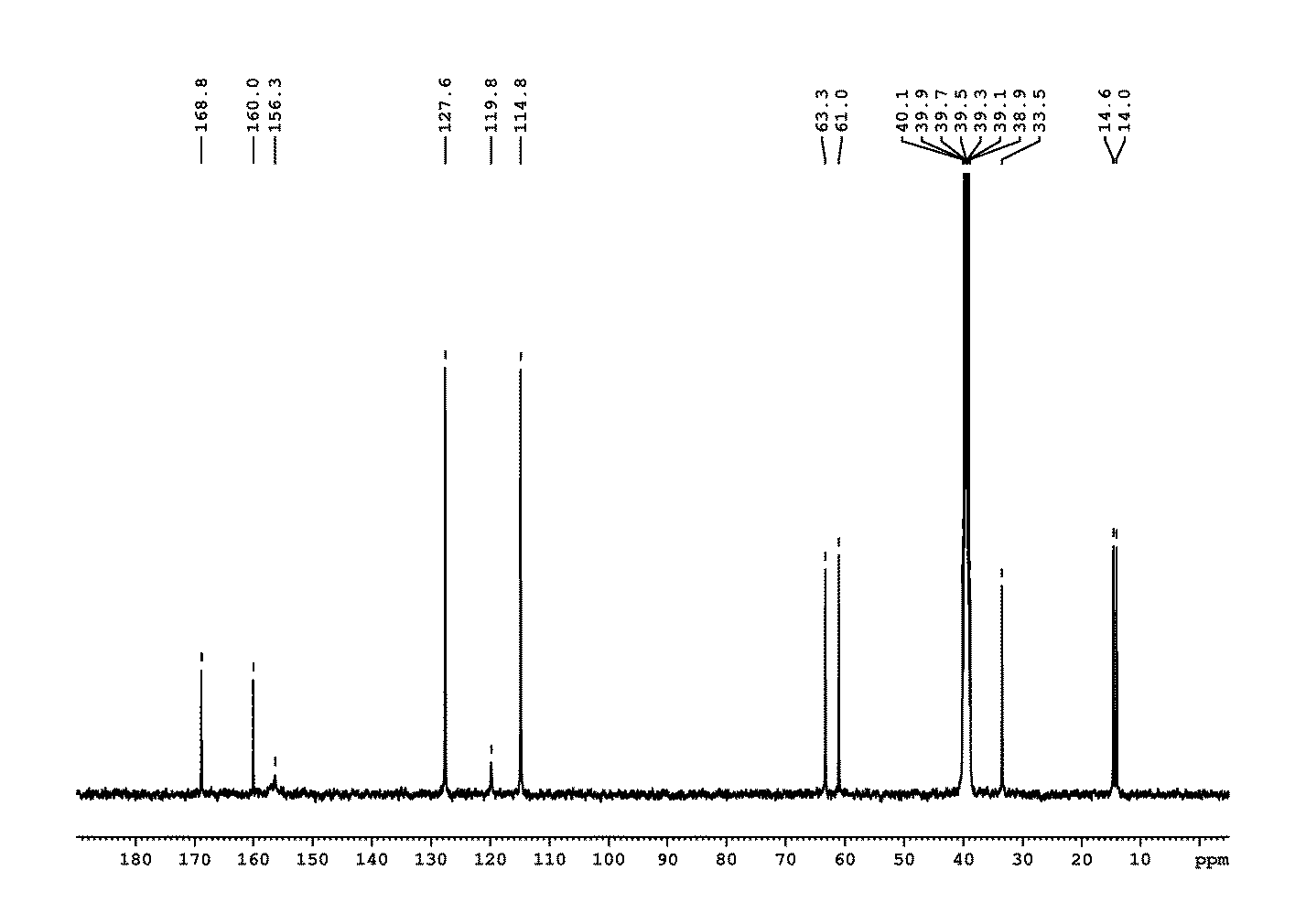
**Figure 15. 13C-MNR spectra of compound1*H*-3-(4-ethoxyphenyl)-5-ethoxycarbonyl-methylsulfanyl-1,2,4-triazole (TZ 53.11)

**
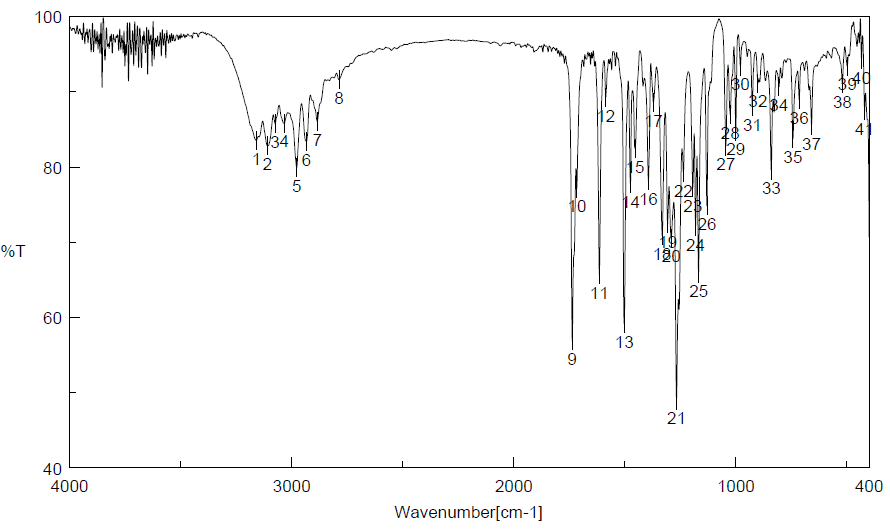
**
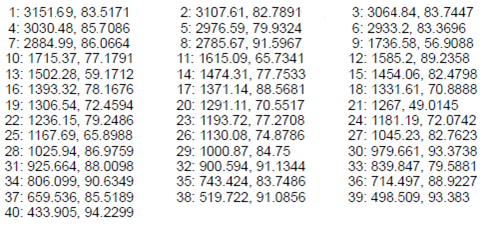


Figure 16. FTIR spectra of compound1*H*-3-(4-n-butoxiiphenyl)-5-ethoxycarbonyl-methylsulfanyl-1,2,4-triazole (TZ 55.11)

**
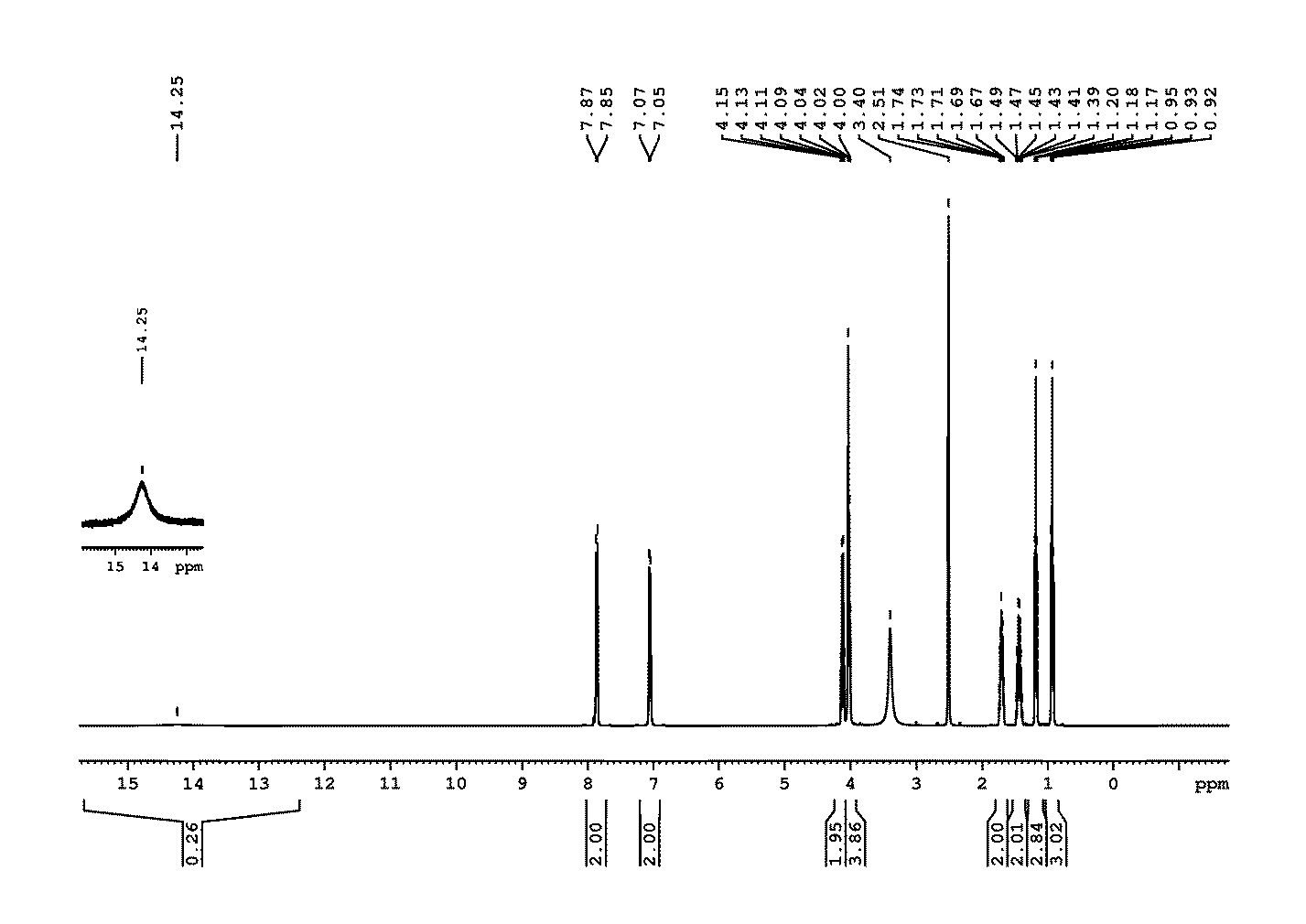
**Figure 17. 1H-MNR spectra of compound1*H*-3-(4-n-butoxyphenyl)-5-ethoxycarbonyl-methylsulfanyl-1,2,4-triazole (TZ 55.11)

**
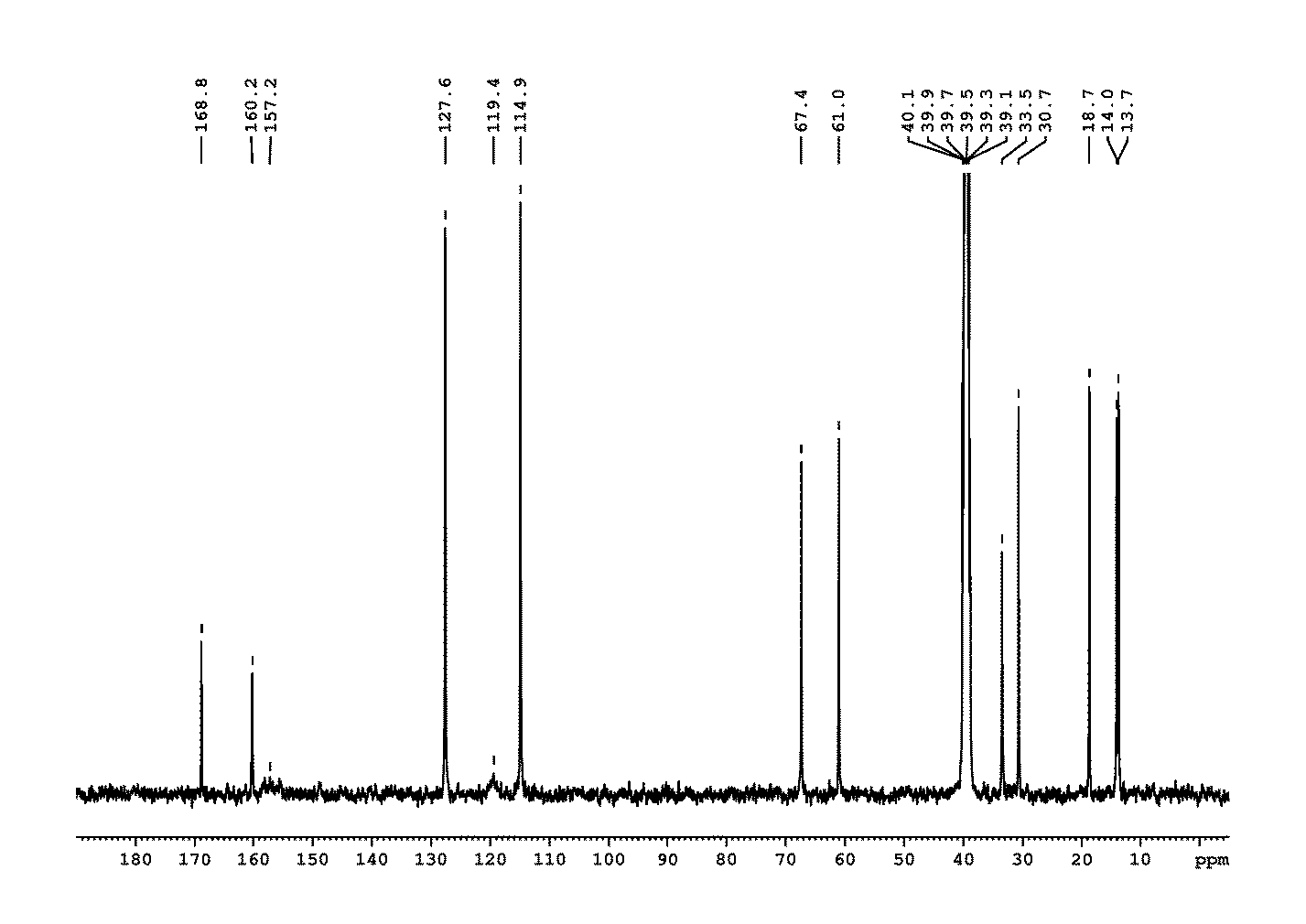
**Figure 18. Spectra a ^13^C-RMN compusului 1*H*-3-(4-n-butoxyphenyl)-5-ethoxycarbonyl-methylsulfanyl-1,2,4-triazole (TZ 55.11)
